# Non-malignant respiratory epithelial cells preferentially proliferate from resected non-small cell lung cancer specimens cultured under conditionally reprogrammed conditions

**DOI:** 10.18632/oncotarget.14366

**Published:** 2016-12-29

**Authors:** Boning Gao, Chunxian Huang, Kemp Kernstine, Vasiliki Pelekanou, Yuval Kluger, Tingting Jiang, Jennifer R Peters-Hall, Melissa Coquelin, Luc Girard, Wei Zhang, Kenneth Huffman, Dwight Oliver, Fumi Kinose, Eric Haura, Jamie K Teer, Uwe Rix, Anh T Le, Dara L Aisner, Marileila Varella-Garcia, Robert C Doebele, Kyle R Covington, Oliver A Hampton, Harsha V Doddapaneni, Joy C Jayaseelan, Jianhong Hu, David A Wheeler, Jerry W Shay, David L Rimm, Adi Gazdar, John D Minna

**Affiliations:** ^1^ Hamon Center for Therapeutic Oncology Research, University of Texas Southwestern Medical Center, Dallas, Texas, USA; ^2^ The Simmons Comprehensive Cancer Center, University of Texas Southwestern Medical Center, Dallas, Texas, USA; ^3^ Department of Pharmacology, University of Texas Southwestern Medical Center, Dallas, Texas, USA; ^4^ Division of Thoracic Surgery, University of Texas Southwestern Medical Center, Dallas, Texas, USA; ^5^ Department of Pathology, Yale University, New Haven, CT, USA; ^6^ Interdepartmental Program in Computational Biology and Bioinformatics, Yale University, New Haven, CT, USA; ^7^ Department of Cell Biology, University of Texas Southwestern Medical Center, Dallas, Texas, USA; ^8^ Department of Pathology, University of Texas Southwestern Medical Center, Dallas, Texas, USA; ^9^ Department of Thoracic Oncology, H. Lee Moffitt Cancer Center and Research Institute, Tampa, Florida, USA; ^10^ Department of Biostatistics and Bioinformatics, H. Lee Moffitt Cancer Center and Research Institute, Tampa, Florida, USA; ^11^ Department of Drug Discovery, H. Lee Moffitt Cancer Center and Research Institute, Tampa, Florida, USA; ^12^ Department of Medicine, Division of Medical Oncology, University of Colorado Anschutz Medical Campus, Aurora, Colorado, USA; ^13^ Department of Pathology, University of Colorado Anschutz Medical Campus, Aurora, Colorado, USA; ^14^ Human Genome Sequencing Center, Baylor College of Medicine, Houston, Texas, USA; ^15^ Department of Internal Medicine, University of Texas Southwestern Medical Center, Dallas, Texas, USA

**Keywords:** conditionally reprogrammed cells, respiratory epithelial cells, non-small cell lung cancer, rock inhibitor, cell culture

## Abstract

The “conditionally reprogrammed cells” (CRC) method, using a Rho kinase inhibitor and irradiated mouse fibroblast cells has been described for the efficient growth of cells from malignant and non-malignant samples from primary tumor and non-malignant sites. Using the CRC method, four institutions independently cultured tumor tissues from 48 non-small cell lung cancers (NSCLC, mostly from primary resected tumors) and 22 non-malignant lungs. We found that epithelial cells could be cultured from tumor and non-malignant lung. However, epithelial cells cultured from tumors had features of non-malignant respiratory epithelial cells which include: 1) among 22 mutations found in the original tumors only two mutations were found in the CRC cultures with reduced frequency (31% to 13% and 92% to 15% from original tumor and CRC culture respectively); 2) copy number variation was analyzed in 9 tumor and their CRC cultures and only diploid patterns were found in CRC cultures; 3) mRNA expression profiles were similar to those of normal respiratory epithelial cells; and 4) co-culture of tumor and non-malignant lung epithelial cells resulted in mostly non-malignant cells. We conclude that CRC method is a highly selective and useful method for the growth of non-malignant respiratory epithelial cells from tumor specimens and only occasionally do such CRC cultures contain a small subpopulation of cancer cells marked by oncogenic mutations. While our findings are restricted to resected primary NSCLC, they indicated the necessity to fully characterize all CRC cultures and the need to develop culture technology that facilitates the growth of primary lung cancers.

## INTRODUCTION

Lung cancer is the leading cause of cancer death for men and women worldwide (http://www.cdc.gov/cancer/international/statistics.htm) [1]. Preclinical human and mouse models of lung cancer are needed to develop new therapeutic and diagnostic approaches and to functionally validate the large number of genetic and epigenetic abnormalities being discovered by a variety of genome wide analytic approaches. Human lung cancer lines, xenografts (including patient derived xenografts, PDXs or cell line derived xenografts, CDXs), and immortalized non-malignant respiratory epithelial cell cultures have been used widely for the study of lung tumor biology and testing of new therapies [2].

We have established over 300 lung tumor cell lines including small cell lung cancer and non-small cell lung cancers (NSCLC) and cultured over 100 non-malignant respiratory epithelial cells (HRECs) including human bronchial epithelial cells (HBECs) and human small airway epithelial cells (HSAECs) in our laboratory over the past decades [3, 4]. Most of these lung cancer cell lines were established from specimens collected at metastatic sites such as the lymph nodes and bone marrow or in pleural effusions [5]. This is because the success rate for establishing lung tumor cell lines from resected primary lung tumors is very low (< 10%) due to the over-growth of fibroblast cells and the senescence of the tumor cells when using “conventional” *in vitro* culture conditions (standard tissue culture medium supplemented with fetal bovine serum, un-published data from the laboratories of Adi Gazdar and John Minna). Likewise the success rate of establishing PDX's from primary lung cancers is only 25–50% at best [6]. In addition, the ability to start continuous cultures from such PDX specimens is also low (un-published data from the laboratory of John Minna). To improve the success rate of non-small cell lung cancer cultures, we established, and routinely use a defined media, ACL4 supplemented with 5% fetal bovine serum [7, 8]. For HBEC and HSAEC cultures, we found they became senescent with growth stopping after ~50 days of *in vitro* culture in defined media (KSFM, Life Technologies for HBECs, and SAGM, Lonza for HSAECs), while exogenous expression of hTERT and CDK4 in HBECs or HSAECs conferred immortality and allowed reproducible establishment of continuously growing non-malignant lung epithelial cell cultures in defined media from many of the specimens [4].

Todaro and Green established 3T3 fibroblast cell lines from the embryos of mice in 1963 and it was later discovered that lethally irradiated 3T3 cells could be used as feeder layers for the long-term culture of epidermal keratinocytes [9–11]. The use of irradiated 3T3 cells for the culturing of human normal and tumor epithelial cells was subsequently reported [12, 13]. Liu et al. published a method of culturing tumor and normal epithelial cells using a Rho kinase inhibitor (ROCKi) Y-27632 and irradiated mouse 3T3 fibroblast feeder cells which they refer to as the “conditionally reprogramed cells” (CRC) method [14]. These authors reported that non-malignant as well as tumor epithelial cells from different organ sites proliferated indefinitely while the growth of accompanying fibroblasts and related stromal cells in tumor tissues were inhibited under CRC culture conditions. If the CRC methods could be applied to all common epithelial malignancies, this would be a very attractive method for the establishment of new human tumor and non-tumor epithelial cultures and cell lines. In addition, this method would represent a particularly significant technical advance to establish cultures from small patient tumor biopsy specimens, which would help in developing “personalized medicine”, based on tests of these tumor cultures, for individual patients.

The goal of our study was to test the ability of the CRC method to establish non-malignant and tumor cell lines from primary resected NSCLCs. We report the cellular and molecular characterization of CRC cultured lung tumor and non-malignant epithelial cells conducted independently in four cancer centers (UT Southwestern, Yale University, Moffitt Cancer Center and University of Colorado). These studies showed that we were able to routinely culture lung epithelial cells but not tumor cells from such primary tumor specimens.

## RESULTS

### Patient information

Patient information is summarized in Table 1 and details provided in Supplementary Tables S1 and S2. Forty-six specimens were collected from resected primary lung cancers and the majority of these specimens were from subjects with early stage NSCLC and two specimens were from NSCLC tumor biopsies. As shown in Supplementary Table S1, with detailed histologic examination, all of the tumor tissues submitted for CRC culture contained tumor cells, except in one case (HCC4084), which was used subsequently as non-malignant CRC culture. On average, there were ~47% tumor cells in the tumor specimens (Supplementary Table S1). A H&E photomicrograph demonstrating the presence of tumor and non-malignant respiratory epithelial cells in a representative lung tumor specimen is illustrated (Supplementary Figure S1).

**Table 1:** Summary of the patient information

	UTSW	Moffitt	Yale	Colorado	Total
Number of patients	20	9	17	2	48
Average age (range)	69.6 (43–86)	75.2 (69–82)	67.4 (32–89)	44.5 (39–50)	68.8 (32–89)
Female/Male	13/7	4/5	7/10	0/2	24/24
KRAS mutation	3	6	4	0	13
EGFR mutation	6	1	2	1	8
PTEN mutation	NA	1	1	N/A	2
ALK rearrangement	NA	0	0	1	1
TP53 mutation	NA	2	NA	NA	2
APC mutation	NA	1	NA	NA	1
Stage IA & IB	9	4	10	0	23
Stage IIA & IIB	10	1	6	0	17
Stage IIIA & IIIB	1	4	1	0	6
Stage IV	0	0	0	2	2
Adenocarcinoma	12	5	6	2	25
Squamous cell carcinoma	4	2	8	0	14
Other types^a^	4	2	3	0	9

Abbreviations: ^a^Other tumor types include carcinoid, neuroendocrine carcinoma, large cell carcinoma, NSCLC not otherwise specified and tumors with mixed squamous/adenocarcinoma types. N/A: not available.

### CRC culture method extends the cultured lifespan of non-malignant respiratory epithelial cells and maintains their ability to differentiate

**Figure 1:**
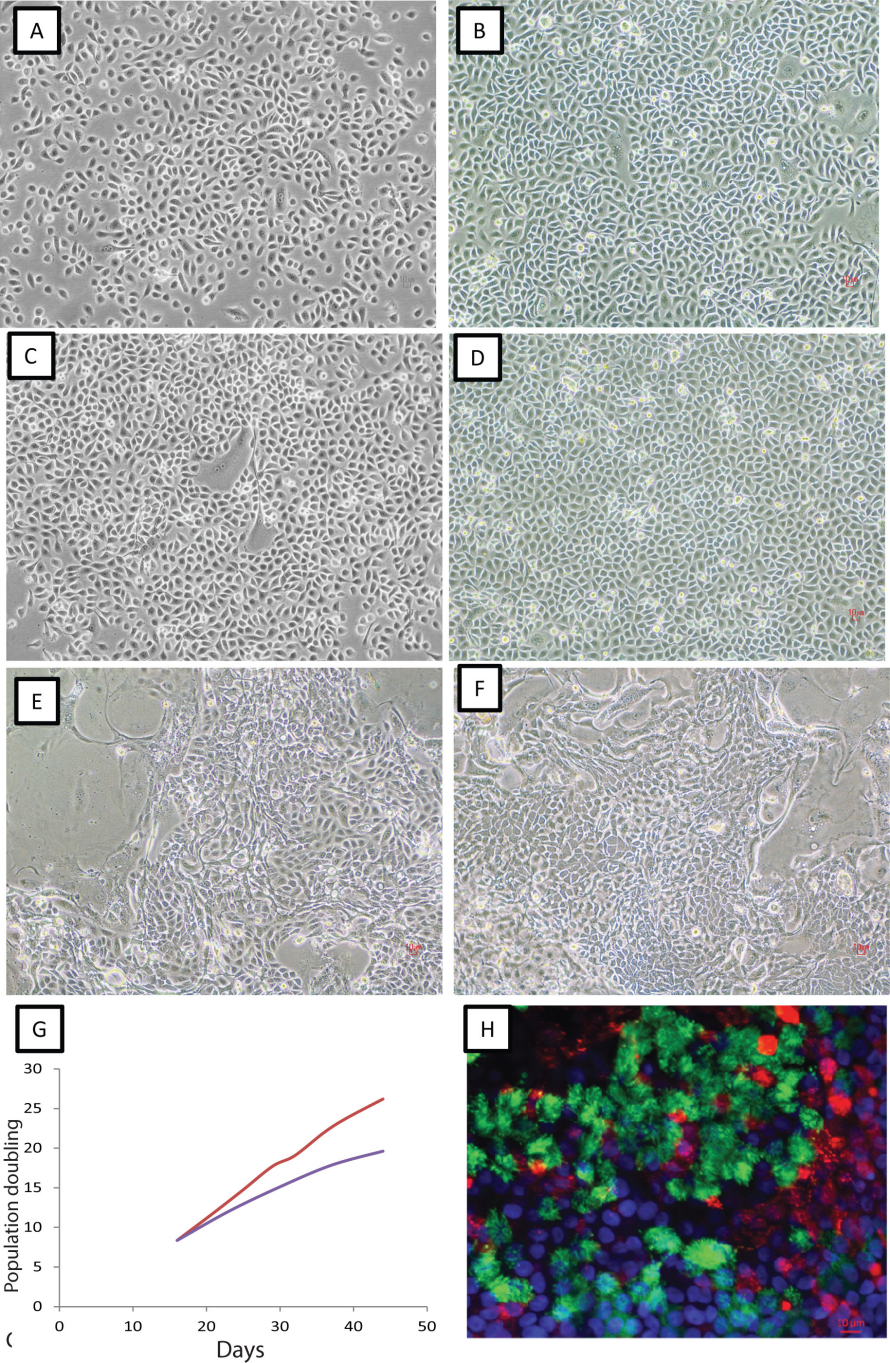
Growth of lung epithelial cells under CRC and non-CRC conditions Representative photomicrographs (original magnification 10×) of non-malignant bronchial epithelial cells (**A** and **B**), small airway epithelial cells (**C** and **D**) and lung tumor specimens (**E** and **F**) cultured under non-CRC (A and C) and CRC (B and D–F) conditions. Cells from A to E are from the same individual. (**G**) Growth rate of normal human bronchial epithelial cells under CRC (red line) and non-CRC conditions (blue line). (**H**) Immunofluorescent staining of differentiation of CRC non-malignant cells cultured under air liquid interface condition. Red: mucin 5B (goblet cells); Green: acetylated α-tubulin (cilia). Blue: DAPI staining of nuclei. Scale bar: 10 nm.

Twenty-two non-malignant resected lung specimens collected from some of the same subjects as part of curative intent resections and apparently free of tumor cells were cultured under CRC conditions. As shown in Supplementary Table S2, all of the non-malignant tissue samples were collected from either peripheral lung or bronchus distant from the corresponding tumors. Epithelial cells grew from all 22 of the non-malignant tissues under CRC conditions. As shown in Figure 1, cells formed dense epithelial cell colonies under both CRC and non-CRC conditions. Cells grew as monolayer cultures, were homogeneous in cell shape and size, and displayed contact inhibition properties under both culture conditions. There was no significant difference in morphology and in genome-wide RNA expression profiles between cells cultured under CRC or non-CRC conditions (using defined medium, see methods) (Figure 1 and Supplementary Figure S5). However, the growth of these non-malignant lung epithelial cells stopped after ~50 days under non-CRC conditions consistent with our previous report [4] while such cells grew robustly for 150 days or longer cultured under CRC conditions (Figure 1). We have previously shown that non-malignant lung epithelial cells cultured under conventional culture conditions can differentiate to ciliated cells and mucous producing cells in ALI (air-liquid interface) culture [15]. We found CRC cultured non-malignant respiratory epithelial cells also differentiated to ciliated cells and mucus producing cells in ALI culture (Figure 1). Thus, we reproducibly found that CRC cultured non-malignant lung epithelial cells maintained pluripotent properties allowing them to fully differentiate and the population doubling lifespan of these lung epithelial cells was dramatically extended as described by Liu et al. [14, 16].

### The morphology of cells from resected primary lung tumor is heterogeneous under CRC culture conditions

Forty-five specimens from resected primary lung cancers and two lung biopsy specimens from late stage lung cancers, all histologically proven to contain tumor cells, were cultured under CRC conditions for an average of 23 days (Supplementary Table S1) and were harvested for molecular studies. We found epithelial cells grew from all tumor tissues we cultured. We observed colonies in the culture of differing morphologies. Some colonies were more “tumor-like” with cells exhibiting different shapes and sizes (Figure 1). However, the vast majority of colonies contained cells with similar shapes and sizes resembling the morphology of normal epithelial cells shown in previously published work [12]. There was no apparent difference in cell morphology depending on the stage of the tumor from the patient. By contrast to lung tumor tissues cultured under conventional conditions where stromal fibroblasts were routinely detected, such fibroblast-like cells were absent in CRC cultures, a finding consistent with the observation made by Liu et al. [14].

### Cells grown from CRC cultured tumor specimens usually lack mutations found in the original tumor specimens

**Figure 2:**
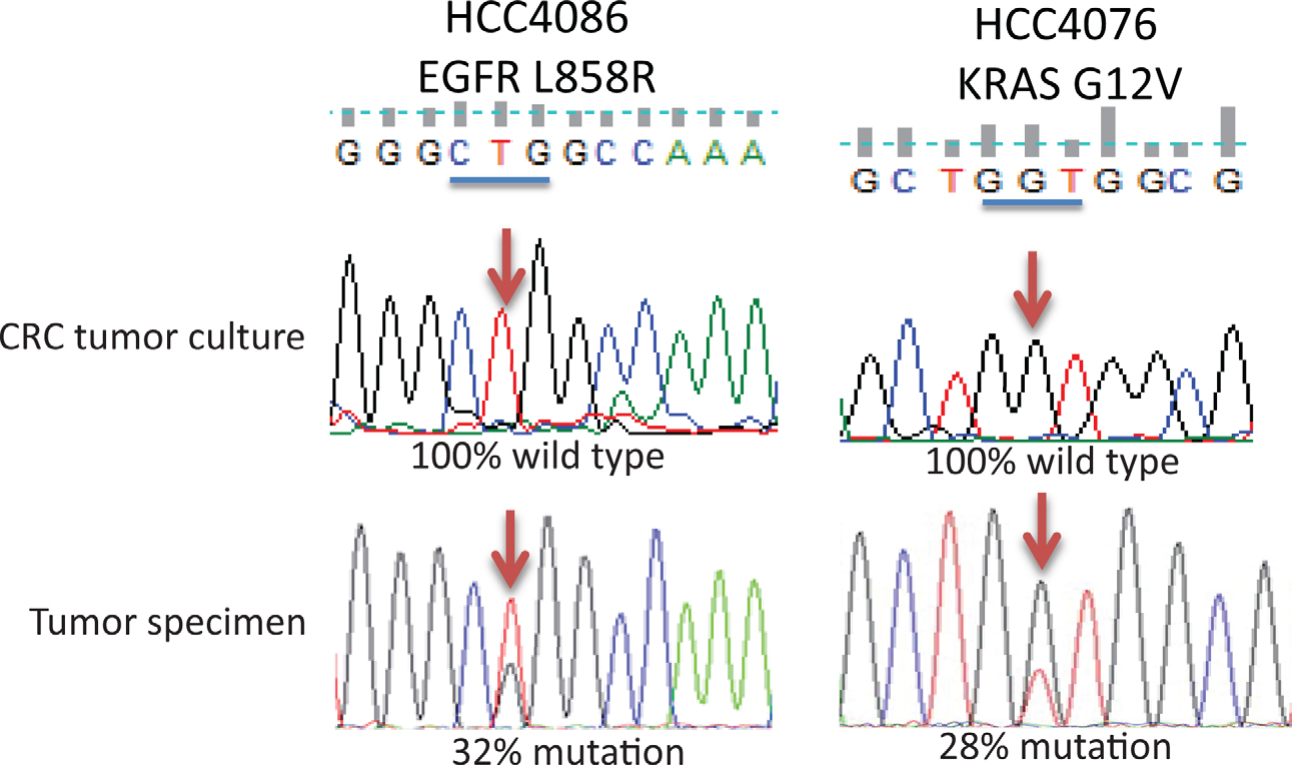
Representative sequencing profiles of oncogenic mutation detected in tumor and cells derived from CRC cultured tumor specimens by target sequencing Upper and lower figure in each panel represents the sequencing profile of lung tumor specimens cultured under CRC conditions and in original tumor specimens respectively. Blue bars indicate the codon where the mutation occurs. Arrows indicate the nucleotide where the mutation occurs.

Eighteen CRC tumor cultures were from NSCLC specimens with a total of 22 known oncogene mutations such as KRAS, EGFR, PTEN, TP53 or ALK rearrangements demonstrated in the primary tumor specimens at diagnosis (Supplementary Table S1). The most straight forward approach to characterize the cultured cells from tumor tissue grown under CRC conditions is to check if these cells carry the same mutations as these found in the tumor. Two methods were used to detect these mutations. One method was to do target sequencing while the other method was to perform exome sequencing. We used the target sequencing approach to detect 22 mutations (mutations were from available clinical data from specimens collected at UTSW, Moffitt and UC) in CRC cultures and compared the mutation status with the mutations detected in original tumor specimens at diagnosis. There was no detectable mutation in these genes except in two cases (HCC4087 and S13-013209) where the mutation rates were decreased from 33% to 13% and from 92% to 15% in the original tumor and in the CRC cultures respectively (Table 2). Two representative sequencing profiles are shown in Figure 2 and the rest of the sequencing profiles are shown in Supplementary Figures S2 and S3. Detailed study of HCC4087 CRC cultured cells showed that the mutant allele frequency dropped from 31% in the original tumor specimen to 13% when cells were cultured under CRC conditions. The mutant allele frequency was increased to 51% when the culture conditions were switched to non-CRC conditions (standard tissue culture ACL4 with 5% serum). As a control, the HCC4087 tumor cell line established using non-CRC condition had a KRAS mutant allele frequency of 67% (Supplementary Figure S2).

**Table 2:** Oncogenic mutations detected in tumor tissue and CRC cultured tumor specimens by target sequencing method

Tumor ID	Tumor mutation	Frequency (%) in tumor specimen	Frequency (%) in CRC cultured tumor specimen
HCC4086	EGFR L858R	32	NP
HCC4117	EGFR Exon 19 Del	50	NP
HCC4119	EGFR L858R	15	NP
HCC4085	EGFR L858R/T790M	40/50	NP/NP
HCC4079	EGFR Exon 19 Del	42	NP
HCC4082	KRAS G12D	27	NP
HCC4076	KRAS G12V	28	NP
HCC4087	KRAS G13C	31	13
MCC-L006T	EGFR frameshift/truncation	6	NP
MCC-L009T	KRAS G12V	19	NP
MCC-L011T	KRAS G13D	15	NP
MCC-L014T	PTEN R130Q/TP53 K132R	4/19	NP/NP
MCC-L018T	KRAS G12D/APC G1116R/TP53 R280I	30/13/14	NP/NP/NP
MCC-L019T	KRAS Q61L	56	NP
MCC-L020T	KRAS G13D	13	NP
MCC-L026T	KRAS G12C	9	NP
S14-005555	EGFR Exon 20 ins	44	NP
S13-013209	ALK rearrangement	92	15^a^

Abbreviations: NP: not present/not detected. ^a^see detailed data in Supplementary Figure S3.

The lack of mutations in KRAS, EGFR and ALK genes in CRC cultured tumor specimens suggested that the cells growing in CRC conditions are mostly non-malignant. However, it is possible that other driver mutations exist in the CRC cultured cells. To further confirm the non-malignant nature of the CRC cultured tumor specimens, we performed exome sequencing on 18 of the CRC cultured tumors cells. Among those, the sequencing data from 9 CRC cultured tumors cells was compared with the sequencing data from original tumor and normal tissue from the same individual (specimens cultured at Yale) and the sequencing data from 7 CRC cultured tumors cells was compared with the normal cells from the same individual and 2 CRC cultured tumor cells were compared with tumor and normal cells from the same individual (specimens cultured at UT Southwestern). We focused on the genes that are commonly mutated in lung cancer reported in the Cancer Genome Atlas (TCGA) studies (Supplementary Table S3) [17, 18]. There were a total of 31 non-synonymous single nucleotide variations (SNVs) with 25 from the original tumors with the mutation fractions of 33% and 6 from CRC cultured tumor specimens with the mutation fractions of 15% (Table 3). As shown in Table 3, the lower KRAS mutation rate was detected in CRC cultured HCC4087 by exome sequencing as we did in target sequencing (Table 2 and Supplementary Figure S2). In addition, we detected a frameshift mutation in TP53 gene (T81fs) in HCC4087 cell line with a frequency of 91% while such mutation was not detected in the HCC4087 CRC cultures (Table 3). Thus, exome sequencing further supported the hypothesis that CRC cultured tumor specimens contain almost exclusively cells not derived from the corresponding tumor specimen.

**Table 3:** SNVs detected in tumor and CRC cultured tumor specimens by exome sequencing

Specimen ID	Tumor type	Gene	Protein alteration	Mutation Frequency	
				Tumor tissue	CRC tumor cultures
SA3	Ad	HRAS	G13D	NP	0.06
SA33	Ad	ARID1A	S958F	0.24	NP
		MGA	D2124N	0.39	NP
		NOTCH1	R353C	0.31	NP
		RIT1	V143L	0.18	NP
		ROS1	P620L	0.44	NP
		TP53	I63F	0.36	NP
		TP53	L62F	0.28	NP
		TP53	Q60X	0.37	NP
SA45	SCC	PTEN	Q214X	0.12	NP
		RB1	L335X	0.10	NP
		RET	G435V	0.13	NP
		TP53	T23P	0.26	NP
SA70	SCC	EGFR	G601E	0.13	NP
		SMARCA4	L968F	0.31	NP
		SMARCA4	A1506T	0.26	NP
		TP53	S109Y	0.34	NP
		NOTCH1	Q2409R	NP	0.07
SA75T	LCC	KRAS	G12V	0.33	NP
		STK11	I303F	0.92	NP
SA77	Ad	RIT1	M54I	0.03	NP
		STK11	E396X	0.64	NP
		NOTCH1	G481V	NP	0.18
		PTEN	P387L	NP	0.15
SA80T	Ad	KRAS	G12A	0.27	NP
		SETD2	S403N	0.08	NP
		RBM10	N149S	0.17	NP
HCC4082	Ad	NOTCH1	Y737X	NP	0.24
HCC4087	Ad/SCC	TP53	T81fs	0.91	NP
		KRAS	G13C	0.72	0.15
Average				0.33	0.15

Abbreviations: NP: not present/not detected. LCC: large cell carcinoma. SCC: squamous cell carcinoma.

### Cells grown from tumor specimens cultured under CRC conditions have diploid genomes

Lung cancer cells are almost always aneuploid with frequent gene amplifications and deletions (TCGA Research Network: http://cancergenome.nih.gov/). We compared the copy number variations (CNVs) detected in 9 lung cancer tissues and the corresponding epithelial cells cultured from these tumors under CRC conditions based on the exome sequencing data. As shown in Supplementary Figure S4, there were frequent CNV in all tumor tissues except in tumor specimen SA59 which was from a 32 years old woman diagnosed with a typical carcinoid tumor (Supplementary Table S1). However, no CNVs were detected in CRC cultured cells from tumor specimens except for a low level gain of chromosome 19 (2.4 copies in average compared with the normal of 2 copies) in all of the nine CRC cultured tumor tissues. Thus, lung cancer tissue cultured under CRC conditions yielded cells with diploid DNA.

### The expression profiles of cells grown from tumor specimens cultured under CRC conditions are similar to non-malignant respiratory epithelial cells cultured under CRC or non-CRC conditions

We performed whole transcriptome mRNA expression analysis to compare tumor specimens cultured using the CRC method, lung tumor cell lines cultured under standard tissue culture conditions, and non-malignant lung respiratory epithelial cells cultured under CRC and non-CRC conditions (as listed in Supplementary Table S4). All microarray data for CRC cultured cells were from UT Southwestern or from Yale, with data analyzed in a blinded fashion. We found that cells derived from tumor specimens cultured under CRC conditions exhibited mRNA profiles that clustered with non-malignant respiratory epithelial cells cultured under CRC and non-CRC conditions rather than with lung tumor cell lines in a non-supervised clustering analysis (Supplementary Figure S5). One of the “tumor” specimens (HCC4084), which was found retrospectively to contain no tumor cells, clustered with the mRNA derived from cells from tumor specimens cultured under CRC conditions. In addition, cells grown from specimens SA3 (tumor specimen) and SA4 (non-malignant specimen) from the same patient were clustered closely to each other. Cells derived from other paired tumor (SA6) and non-malignant tissue (SA7) cultured under CRC conditions also clustered closely to each other.

Using the mRNA expression data we identified 50 genes that were the most up or down regulated comparing cells from lung tumor specimens cultured under CRC conditions with a large panel of known lung cancer cell lines (Supplementary Table S5). Genes up-regulated in cells from tumor specimens grown under CRC culture conditions are enriched with markers for lung basal cells such as TP63 (> 60 fold), podoplanin (PDPN, > 36 fold), cytokeratin genes KRT6A (> 729 fold), KRT6B (> 98 fold), and adhesion molecules LGALS7B (> 200 fold). By contrast, many lung cancer specific genes were down-regulated in the cells from CRC cultured tumor specimens. Examples of down-regulated genes include EEF1A2 (< 60 fold) which is highly expressed in many tumor types [19], XAGE1A, a well-known tumor/testis antigen gene (down-regulated < 30 fold) [20] and pleiotrophin (PTN), which is highly expressed in small cell and NSCLC, was down-regulated 16 fold in cells from lung tumor specimens cultured under CRC conditions [21]. Thus, a comparison of gene expression profiles of lung non-malignant cells grown under either CRC or non-CRC conditions, or cells from tumor specimens cultured under CRC conditions showed similar RNA patterns in contrast to lung tumors indicating that CRC conditions preferentially grew non-malignant epithelial cells whether their original source were tumor tissue or non-malignant tissue.

### Normal lung epithelial cells over-grow lung tumor cells under CRC co-culture conditions

That CRC cultured cells from tumor specimens were almost exclusively non-malignant suggested that non-malignant cells preferentially grow under CRC conditions. A normal/tumor cell co-culture experiment was designed to directly compare the growth rate of these cells under CRC condition. We have previously established a lung tumor cell line HCC4017 and we have cultured un-immortalized (UI) primary epithelial cells (HBEC30-UI) from the non-malignant bronchus from the same individual. In addition, we have established a continuously growing cell line HBEC30-KT by immortalizing HBEC30-UI cells with CDK4/hTERT genes. Cells from this individual were chosen because there is a KRAS mutation in tumor cell line HCC4017 while there is no KRAS mutation in HBEC30-KT and HBEC30-UI cells (Supplementary Figure S6) [22]. Thus we were able to use the percentage of KRAS mutations to quantify the amount of HCC4017 cells in the culture. Co-cultures of HCC4017/HBEC30-UI and HCC4017/HBEC30-KT cells were performed separately. Equal number of cells were seeded on irradiated 3T3J2 feeder cells at the same time and cultured under CRC conditions. Cells were harvested 5–11 days later, genomic DNAs were made and Sanger sequencing method was used to identify the sequence of wild type and mutated KRAS genes. As shown in Supplementary Figure S6 and summarized in Table 4, 100% of HCC4017 sequence had KRAS mutation (G34T) while HBEC30-KT and HBEC30-UI had wild type KRAS (G34) when each of the three cells was cultured alone. No KRAS mutation was detected after co-culture of HCC4017/HBEC30-KT for five days. In the HCC4017/HBEC30-UI co-culture, there were less than 10% of cells with KRAS mutation at day seven and no KRAS mutation was detected at day eleven. Thus, in this experimental mixed population we found normal lung epithelial cells to rapidly take over mixed culture of lung cancer and normal lung epithelial cells.

**Table 4:** KRAS mutation detected in HCC4017/HBEC30-KT and HCC4017/HBEC30-UI co-cultures by target sequencing

Cell	Culturing days	KRAS mutation fraction
HCC4017		100%
HBEC30-KT		NP
HBEC30-UI		NP
HCC4017/HBEC30-KT	5	NP
HCC4017/HBEC30-UI	7	< 10%
HCC4017/HBEC30-UI	11	NP

Abbreviations: NP: not present/not detected.

## DISCUSSION

Liu et al. published a method of culturing normal and tumor epithelial cells from different types of tissues, including lung, under CRC culture conditions [14]. They reported normal cells and tumor cells from resected specimens or biopsies can be “reprogrammed” to (basaloid) stem-like cells that proliferate indefinitely under the culture conditions. The CRC culturing method has generated tremendous enthusiasm in the cancer cell biology research community. As of June 2016, using the Scopus search engine, we found 139 publications that cited the CRC method paper (http://www.scopus.com). However, most were review papers acknowledging the newly published CRC method. No detailed characterizations of the majority of CRC cultured cells have been reported.

In this report, four different university cancer centers independently used the CRC method (either original or slightly modified) as described by Liu et al. to establish cultures of lung cancer cells from 46 resected (one without tumor cells) and two biopsies of primary NSCLC specimens. In brief, using the CRC method on NSCLC specimens, we routinely establish continuously replicating epithelial cell cultures which mostly do not have mutations found in the lung cancer specimens from which they were derived, and express mRNA expression profiles similar to large and small airway epithelial cells.

We summarized the culturing conditions and the results from each of the four cancer centers in Supplementary Tables S6 and S7 respectively. While two of the participating cancer centers used the original method described by Liu et al. two used slightly modified methods (Supplementary Table S6). However, the results from all four centers were similar (Supplementary Table S7). In all cases, the cells that grew had properties of non-malignant lung epithelial cells including morphology, lack of tumor derived mutations, diploid copy number and mRNA expression profiles. To directly compare the growth of tumor cells with non-malignant cells, we did tumor/normal cells co-culture experiments using the same feeder layer cells (3T3J2 strain) and medium (F medium) that used by Liu et al. [14]. We found normal lung bronchial epithelial cells, either primary cells or immortalized cells, grow dominantly when co-cultured with the tumor cells from the same individual under the CRC growth conditions. We conclude that non-malignant lung epithelial cells preferentially proliferate under CRC conditions, with variable, predominantly low representation of tumor cell subpopulations. The method also led to the establishment of cultures of lung epithelial cells with similar properties when applied to histologically confirmed, non-malignant lung obtained at the time of the same surgical resections. While all of the centers involved in this study have experience growing human lung cancer in tissue culture, at UT Southwestern Medical Center, researchers have extensive experience with the culture of lung cancers and non-malignant respiratory epithelial cells under non-CRC conditions, which permitted comparison of cells grown under other conditions to cells cultured under CRC conditions [2, 23, 24].

Primary lung epithelial cells are cultured in serum free medium in conventional culture because these cells grow poorly in the presence of serum and undergo senescence [23, 25]. Therefore, the result that non-malignant lung epithelial cells were preferentially proliferating under CRC conditions was unexpected since the culture medium used under CRC conditions was supplemented with 5% fetal calf serum. The fact that these normal-like epithelial cells grew robustly under CRC conditions suggests that serum induced differentiation is inhibited under CRC conditions.

There was a consistent low level of gain of chromosome 19 (2.4 copies on average instead of 2 copies) in all of the CRC cultured tumor cells. In contrast, there was no consistent low level gain of chromosome 19 but there were high levels gain or loss of chromosome 19 in several of the original tumor tissues (SA33, SA70, SA75, SA77 and SA83). This observation is interesting and warrants further investigation.

Three published studies used the modified CRC method for the culturing of lung tumor cells [26–28]. Two of these studies demonstrated the presence of cultured mutation positive tumor cells although no detailed characterization of the cells was reported in the publication [26, 27]. There are two major differences between our study and the two published positive reports: 1) the sources of the starting tumor tissues are different. The majority of the tumor specimens in the two reports were from metastatic tumor cells and fluids (ascites or pleural effusion) and as a result, there are no normal lung epithelial cells present in pleural effusions or in ascites. Thus, there was no overgrowth of normal epithelial cells in their CRC cultures. In contrast, 46 out of 48 of the tumor specimens we studied were from resected lung with variant degree of non-malignant lung epithelial cells in the tumor tissues. In addition, pleural effusion and ascites mostly occur in late stage lung cancers while the resected lung tumors in our studies are mostly from early stage lung cancers. We know from our own experience that tumor cells from metastasis such as pleural effusion grow more robustly than the growth of cells from resected lung tumor in culture based on the 160 consecutive tumors we processed during the past 5 years. 2) The two published studies used modified CRC methods. Both groups used irradiated human fibroblast cells instead of using mouse 3T3 cells that reported by Liu et al. and used by us. In addition, the two groups cultured cells from tumor tissues under CRC conditions (with human fibroblast cells) at the beginning and then switched to use the conventional method (no 3T3 and no Rock inhibitors). It will be of great interest going forward to determine the effect of starting with malignant effusions and metastatic sites as well as using modified CRC method in future work.

In summary, we have found the CRC method is useful for the culture of non-malignant respiratory epithelial cells free of fibroblast contamination from primary lung cancers and accompanying non-malignant lung tissues. However, successful and reproducible growth of tumor cells from resected lung tumor specimens will require modification of current or development of new methodology. We wish to stress, that the ability of the CRC methods to routinely generate continuously growing cultures of lung epithelial cells from malignant as well as non-malignant lung tissues without genetic manipulation is of significant importance to study of the cellular and molecular pathogenesis of lung cancer. Besides further manipulation of such cells (for example by introduction of oncogenic changes), the role of such cells in premalignancy including their effect on tumor cells and the microenvironment will be of great interest. While our study was largely limited to curative intent resections for NSCLC, our findings indicate the necessity to completely characterize CRC cultures from all malignant and non-malignant sources.

## MATERIALS AND METHODS

### Specimen collection

Forty-eight primary NSCLC specimens (46 from subjects undergoing curative intent resections, while two were biopsies) and 22 non-malignant tissues (collected during the period of 2013 and 2014) were used in the study after obtaining Institutional Review Board approval and informed consent. The percentage of tumor cells present in the specimens was estimated by a pathologist in each institute (Supplementary Table S1). Non-malignant lung tissues collected at UTSW were in an area of the lobe furthest away from the tumor. The bronchus was 2–3 cartilaginous rings adjacent to the staple line of resection. Lung tissues collected at Yale were located outside of the tumor margin. The peripheral lung and the bronchus were histologically examined and no tumor tissues were found in the non-malignant tissue collected at both institutes.

### Cell culture

Tumor cell lines with a prefix of NCI-H or HCC were established by us (John Minna and Adi Gazdar at UT Southwestern) and the rest of the tumor cell lines were purchased from ATCC (Manassas, VA). All cells used in the culture were tested mycoplasma free and DNA fingerprint were used for the authentication of each cell. Cells were cultured using standard tissue culture incubator conditions in a humidified atmosphere of 5% CO_2_ at 37°C using our “conventional” methods and the reported CRC method. Our conventional method for the culture of non-malignant HBECs was described previously using KSFM (Life Technologies, Carlsbad, CA) [4]. Non-malignant HSAECs from peripheral lung were cultured in SAGM (Lonza, Portsmouth, NH). Culture media for HBECs and HSAECs are serum free. RPMI 1640 (Sigma, St. Louis, MO) supplemented with 5% FCS were used for the routine culturing of lung tumor cell lines. For CRC cultures, HBECs or HSAECs were added to the irradiated 3T3 cells in the presence of 10 uM ROCK inhibitor Y-27632 (Enzo, Farmingdale, NY) and their respective media. The CRC tumor culture method described by Liu et al. [14] was followed without modification at two institutions (Yale and University of Colorado), while the Swiss 3T3 (J2 strain) cells were replaced by Swiss 3T3 albino strain (ATCC, Manassas, VA) at Moffitt Cancer Center. There are two modifications of the original CRC method at UT Southwestern: 1) Swiss 3T3 NIH/3T3 strain (ATCC, Manassas, VA) cells were used instead of Swiss 3T3 (J2 strain). 2) ACL4 medium which was developed for establishing lung tumor cell lines was used instead of F medium [7, 8]. Both F and ACL4 media are supplemented with 5% fetal bovine serum. Three major additives (Insulin, Hydrocortisone and Epidermal growth factor) in the F medium are present in ACL4 in addition to other additives as described previously [7, 8]. The medium was replaced every 2 days. No individual clones were selected. Culturing days for each cell are indicated in Supplementary Table S1. Methods used in the four institutions are summarized in Supplementary Table S6.

### Co-culture of paired NSCLC HCC4017 and respiratory epithelial HBEC30 cells

Equal number (4000 cells) of adenocarcinoma HCC4017 and corresponding immortalized bronchial epithelial HBEC30-KT or unimmortalized bronchial epithelial cells HBEC30-UI were seeded in a T25 flask with irradiated 3T3J2 cells (purchased from Georgetown University) in the presence of F5 medium and Rock Inhibitor Y-27632 (5 uM). Cells were harvested after 5, 7 or 11 days. Genomic DNAs were prepared and Sanger DNA sequencing method was used to detect the KRAS mutation.

### Air-liquid interface (ALI) cultures and immunofluorescent staining

ALI culture method was described previously [16, 29]. Antibodies to mucin 5B (Santa Cruz (H-300), sc-20119, Dallas TX) and acetylated α-tubulin (clone 6-11B-1, T 6793, Sigma St. Louis, MO) were used at 1:50 dilutions.

### Mutation analysis

Sanger method was used for the target sequencing analysis at UT Southwestern. Information for sequencing primers used for testing CRC tumor cultures was as described by Shigematsu and colleagues [30]. Primers for sequencing tumor specimens were lab developed. The TruSight Tumor 26 Panel (Illumina) was used for generating mutation data at Moffitt Cancer Center and at University of Colorado. FISH (Fluorescence *in situ* hybridization) method (Vysis LSI ALK Break Apart FISH Probe Kit from Abbott Molecular) was used for the detection of ALK rearrangements [31].

Exome sequencing was performed at Yale and Baylor. Sequencing protocol used at Yale is the following: Tumor and normal DNAs used in the exome sequencing were extracted from Formalin-Fixed Paraffin-Embedded tissues. DNA from CRC cultured cells were extracted from the cell culture. The exomes of samples were captured by Nimblegen human solution-capture exome array (SeqCap EZ version 2). The library was sequenced on Illumina (San Diego, CA) HiSeq 2000 in paired-end 75-cycles mode at the Yale Center for Genome Analysis. Reads were filtered by Illumina CASAVA 1.8.2 software, trimmed at the 3’ end using FASTX v0.0.13, aligned to the merged human (GRCh37) and mouse (GRCm38) reference genome by Burrows-Wheeler Aligner v0.7.5a. PCR duplicates were removed by MarkDuplicates algorithm. Local realignment around putative and known insertion/deletion (indel) sites and base quality recalibration were performed using Genome Analysis Toolkit (GATK v3.1.1). We extracted pair-end reads that were only aligned to human genome for mutation calling. MuTect v.1.1.4 and Strelka v.1.0.14 were used to call somatic single nucleotide variants (SNV) and indels, respectively.

Sequencing protocol used at Baylor is the following: DNA samples were constructed into Illumina paired-end pre-capture libraries according to the manufacturer's protocol (Illumina Multiplexing_SamplePrep_Guide_1005361_D) with the below described modifications. Libraries were prepared using Beckman robotic workstations (Biomek FX and FXp models). The complete protocol and oligonucleotide sequences are accessible from the HGSC website (https://www.hgsc.bcm.edu/sites/default/files/documents/Protocol-Illumina_Whole_Exome_Sequencing_Library_Preparation-KAPA_Version_BCM-HGSC_RD_03-20-2014.pdf).

Briefly, 0.5 ug of DNA in 70 ul volume was sheared into fragments of approximately 200–300 base pairs in a Covaris plate with E210 system (Covaris, Inc. Woburn, MA) followed by end-repair (NEBNext End-Repair Module; Cat. No. E6050L), A-tailing (NEBNext^®^ dA-Tailing Module; Cat. No. E6053L) and ligation of the Illumina multiplexing PE adaptors with barcode sequences using the ExpressLink™ T4 DNA Ligase (A custom product from LifeTech). In total, a set of 12 such barcodes were used on these samples. Pre-capture Ligation Mediated-PCR (LM-PCR) was performed for 6–8 cycles using the Library Amplification Readymix containing KAPA HiFi DNA Polymerase (Kapa Biosystems, Inc., Cat # KK2612). Universal primer LM-PCR Primer 1.0 and LM-PCR Primer 2.0 were used to amplify the ligated products. Purification was performed with Agencourt AMPure XP beads were used for post PCR clean up as well as after each enzymatic reaction. Libraries were quantified and their size distribution determined using the LabChip GX electrophoresis system (PerkinElmer) and gel analysis using AlphaView SA Version 3.4 software.

For the hybridization step, four pre-capture libraries were pooled together (~250 ng/sample for a total of 1 ug per pool). These pooled libraries were then hybridized in solution to the HGSC VCRome 2.1 design^1^ (42 Mb, NimbleGen) according to the manufacturer's protocol *NimbleGen SeqCap EZ Exome Library SR User's Guide (Version 2.2)* with minor revisions. Human COT1 DNA and full-length Illumina adaptor-specific blocking oligonucleotides were added into the hybridization to block repetitive genomic sequences and the adaptor sequences. Post-capture LM-PCR amplification was performed using the Library Amplification Readymix containing KAPA HiFi DNA Polymerase (Kapa Biosystems, Inc., Cat# KK2612) with 12 cycles of amplification. After the final AMPure XP bead purification, quantity and size of the capture library was analyzed using the Caliper LabChip GX electroporsis system. The efficiency of the capture was evaluated by performing a qPCR-based quality check on the enrichment level of four standard NimbleGen control loci. Successful enrichment of the capture libraries was estimated to range from a 6 to 9 of ΔCt value over the non-enriched samples.

Sequencing runs were performed in paired-end mode using the Illumina HiSeq 2000 platform. Using the TruSeq SBS Kits (Cat. No. FC-401-3001), sequencing-by-synthesis reactions were extended for 101 cycles from each end, with an additional 7 cycles for the index read. The detailed information was published [32].

### Copy number variation (CNV) analysis

CNV was estimated based on exome sequencing data and analyzed using the exomeCNV package in R using the default setting of tumor/CRC with the paired normal sample. Log2 ratio (CNV) of sample vs control was calculated, where sample was either tumor or CRC and control was the paired normal. The log ratios were adjusted by subtracting their median value so that they were centered around 0. Local averaging was performed by partitioning the data in at most 100 datapoints per partition and using their mean for the segmentation step, which was done using the R package DNAcopy.

### Microarray and un-supervised clustering analysis

Whole transcriptome mRNA expression microarrays were performed on several types of specimens. 1) Lung tumor cell lines were established at UT Southwestern or purchased from ATCC. 2) Tumor specimens cultured under CRC conditions, from UT southwestern and Yale. 3) Accompanying non-malignant lung tissues cultured under CRC conditions (UT Southwestern and Yale). 4) Un-immortalized (UI) human respiratory epithelial cells were established at UT Southwestern.

Illumina Human WG6-V3 or V4 arrays were used for genome-wide expression studies. All arrays were log_2_-transformed and quantile-normalized. For genes with multiple replicate probes, the probe with the highest mean standard deviation was selected Group comparisons were done using minimal fold changes of 2 or 4, with significance assessment by *T*-test. Multiple test corrections were done with the Benjamini-Hochberg method. Hierarchical clustering was performed using average linkage and Pearson correlation distance metric. Microarray data can be found in Gene Expression Omnibus (http://www.ncbi.nlm.nih.gov/geo/) under accession numbers GSE41271 (tumor tissues), GSE32036 (tumor lines, HBECs, HSAECs), GSExxxxx (CRC lines, other HBECs, HSAECs), and GSExxxxx (Yale CRC lines).

## SUPPLEMENTARY MATERIALS FIGURES AND TABLES






