# Supplementary material for: Non-malignant respiratory epithelial cells preferentially proliferate from resected non-small cell lung cancer specimens cultured under conditionally reprogrammed conditions
Source: Oncotarget. 2016 Dec 29;8(7):11114–26. doi: 10.18632/oncotarget.14366 (PMC5355251; doi:10.18632/oncotarget.14366)
Supplement: Supplementary file 1 [file oncotarget-08-11114-s001.pdf]

# Non-malignant respiratory epithelial cells preferentially proliferate from resected non-small cell lung cancer specimens cultured under conditionally reprogrammed conditions

## Supplementary Materials

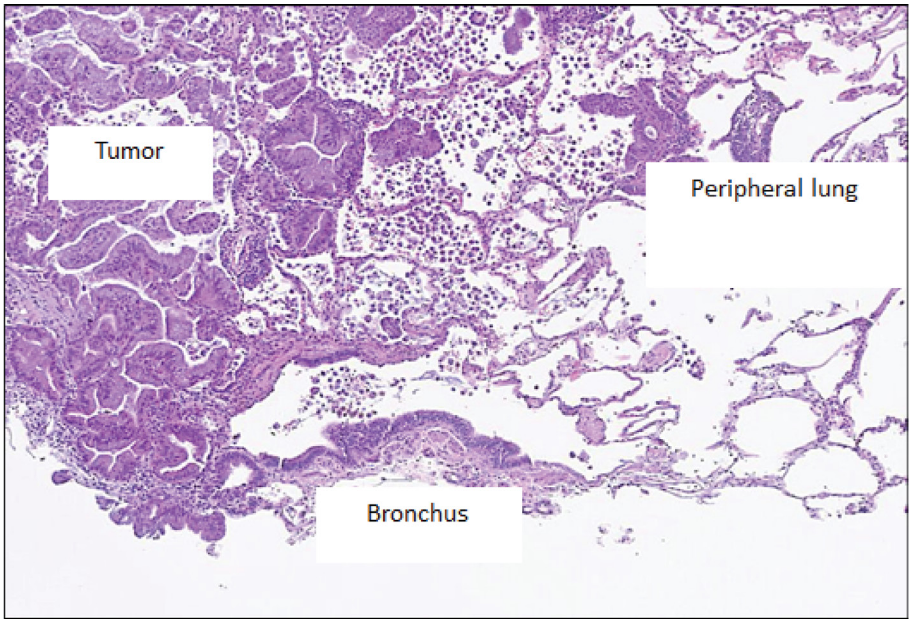


**Supplementary Figure S1: Tumor and normal cells are present in a lung cancer specimen.** Representative H/E staining of a specimen showed tumor cells, a bronchus with hyperplastic bronchial epithelial cells and normal appearing alveolar tissues.

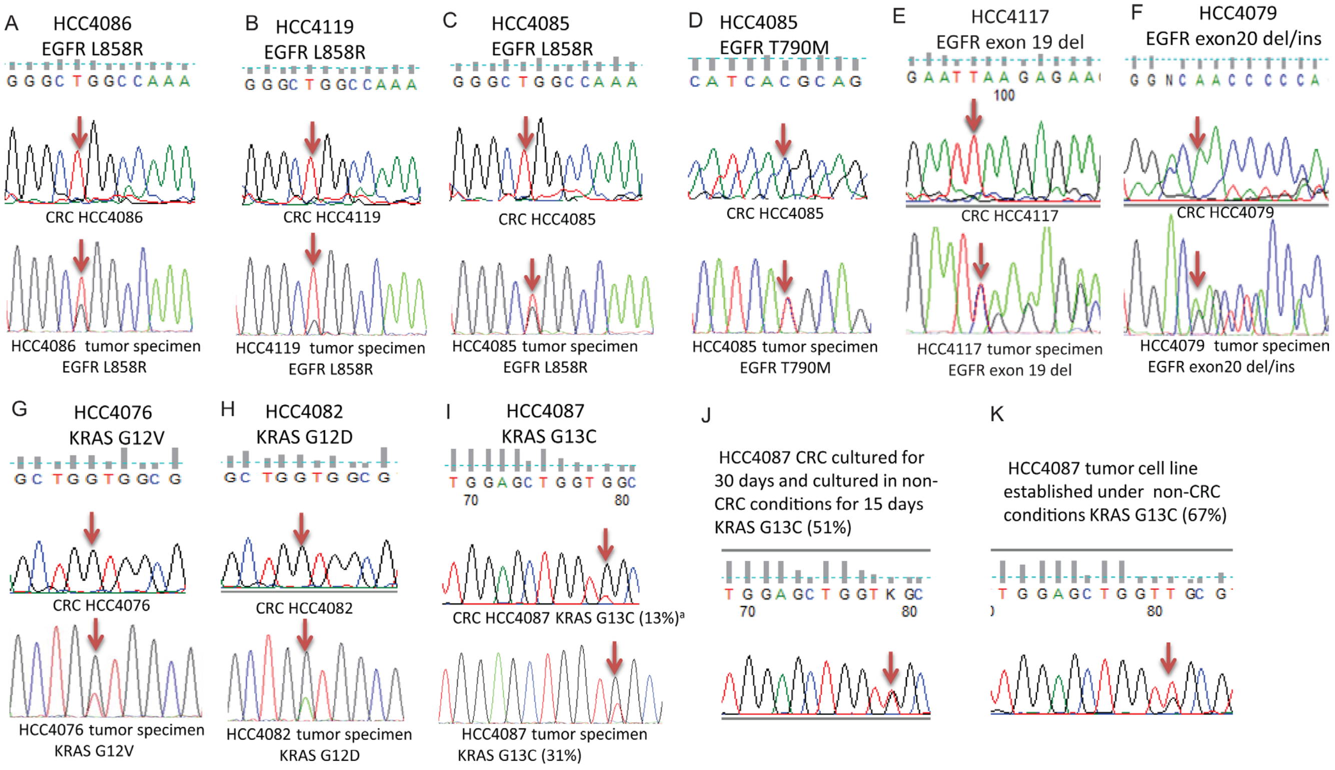


**Supplementary Figure S2: Oncogenic mutations detected in tumor and cells derived from CRC cultured tumor specimens by target sequencing (samples from UT Southwestern).** (**A**–**I**) Upper and lower figure in each panel represents the sequencing profile of lung tumor specimens cultured under CRC conditions and original tumor specimens respectively. (A: HCC4086; B: HCC4119; E: HCC4117, F: HCC4079, G: HCC4076; H: HCC4082 and I: HCC4087) carry one mutation and one tumor carries two mutations (HCC4085 in C and D). Sequencing profile from HCC4087 is shown in panel (**I**–**K**). [a]HCC4087 tumor tissue were cultured for 30 days under CRC conditions. Arrows indicate the mutated nucleotides or nucleotide where insertions or deletions start.

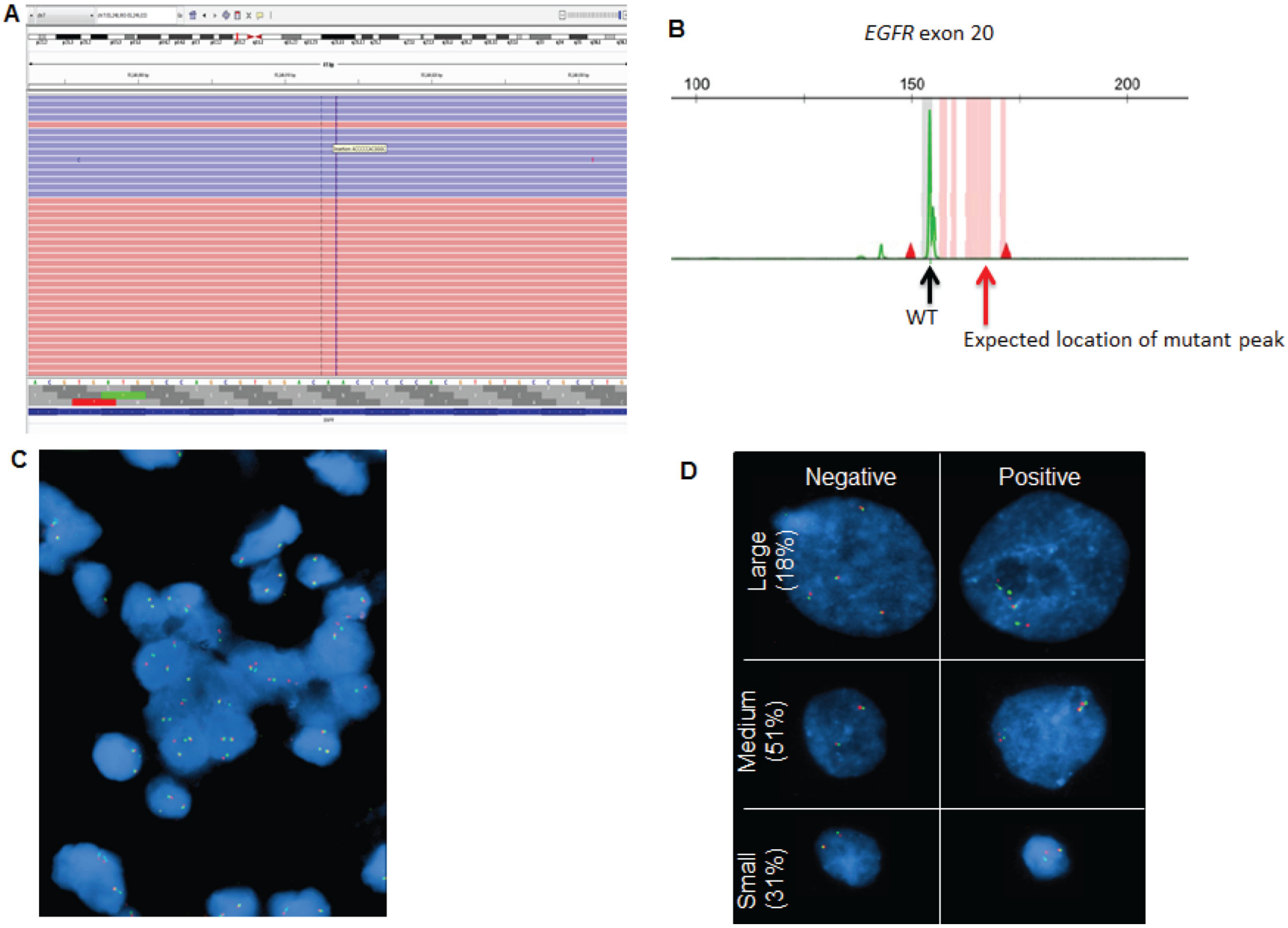


**Supplementary Figure S3: Oncogenic mutation detected in tumor and cells from CRC cultured tumor specimens (Samples from University of Colorado).** (**A**) Targeted dual-strand next generation sequencing performed in a CLIA-certified laboratory demonstrates that the original patient biopsy contains a 12 base pair insertion (c.2313_2314insCCCCACGGGCAC; p.N771_ P772insPHGH) in exon 20 of *EGFR*, as visualized in Integrated Genomics Viewer (Broad Institute). (**B**) PCR-sizing assay performed on cultured cells obtained concurrently from the specimen in panel A demonstrates no detectable presence of *EGFR* exon 20 insertional mutation. Black arrow demonstrates a peak corresponding with wild-type EGFR, and the red arrow indicates the anticipated position of a peak if the 12 base pair insertion originally identified were present. (**C**) ALK FISH in patient tumor sample from a biopsy of a right lung mass demonstrating 92% of cells with positive (split red and green) signals. (**D**) ALK FISH in CRC culture from the same biopsy in (C) by nuclear size demonstrating that the majority of the cells that expanded in culture were small or medium size nuclei, the majority of which were negative for an ALK gene rearrangement. The rate of positive cultured cells for ALK FISH routinely runs significantly higher than tumor sample biopsies, likely due to less nuclear truncation in ALK FISH assays for cultured cells on slides.

## REFERENCE

1. Camidge et al. Cancer. 2012; 118:4486–94. doi: 10.1002/cncr.27411. Epub 2012 Jan 26.

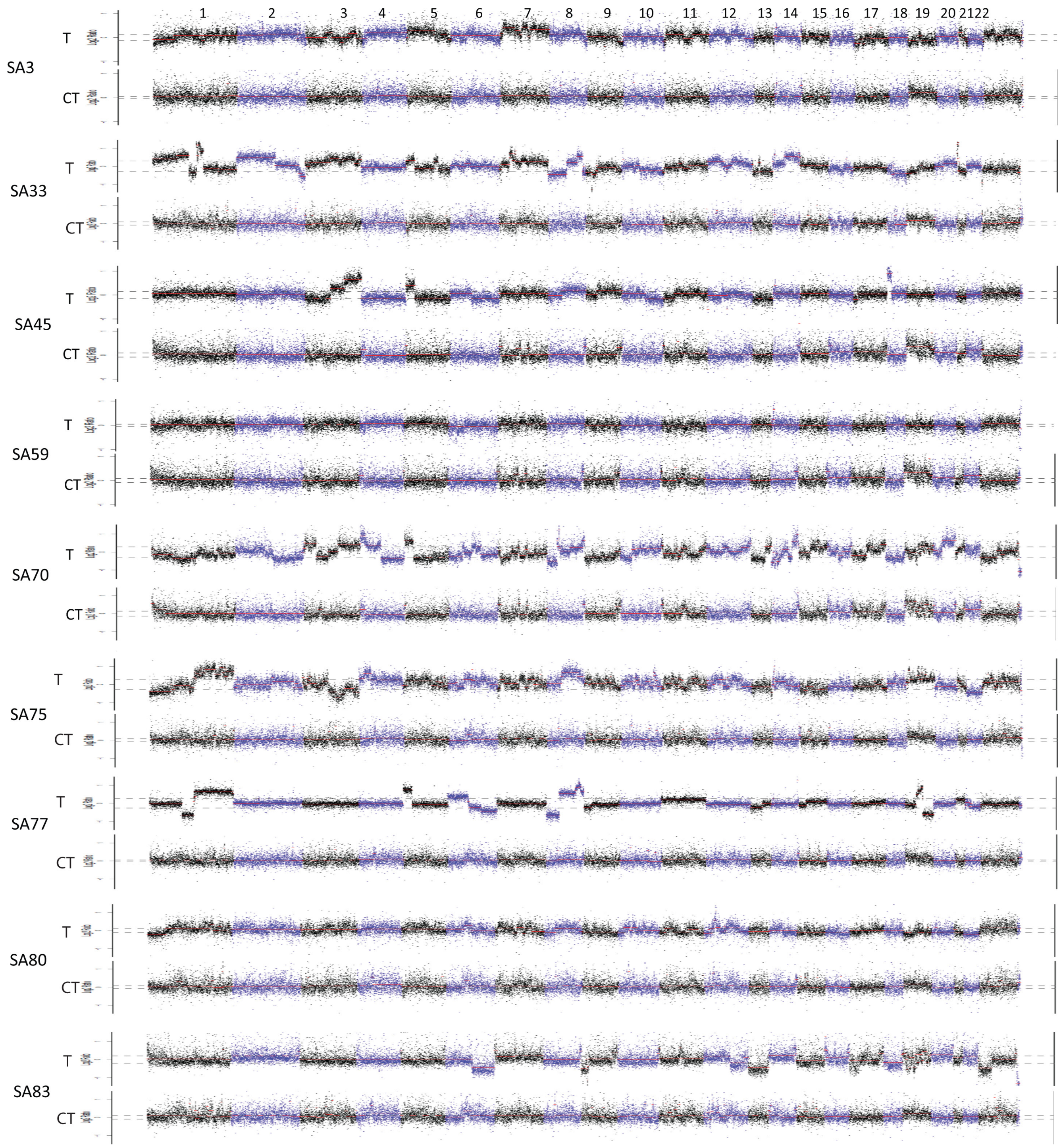


**Supplementary Figure S4: Cells from CRC cultured tumor specimens have diploid genomes.** Alternating blue and black colors from left to right represent somatic chromosomes 1–22. CNV from 9 different lung tumor specimens (T) and the corresponding cultured cells under CRC conditions (CT) are shown.

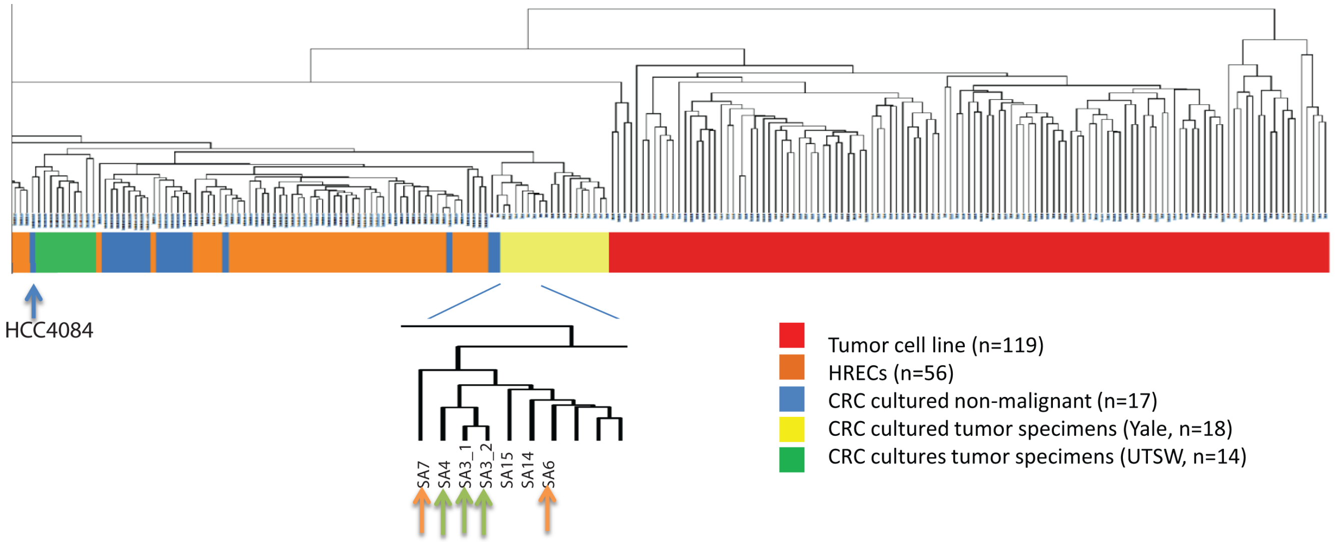


**Supplementary Figure S5: Un-supervised clustering analysis.** RNAs from lung tumor cell lines (red, *n* = 119), unimmortalized primary human respiratory epithelial cells (HREC, orange, *n* = 56), cells from non-malignant tissues cultured under CRC conditions (blue, *n* = 17), cells from lung tumor tissue cultured under CRC conditions at Yale (yellow, *n* = 18) and lung tumor tissue cultured under CRC conditions at UTSW (green, *n* = 14) were used as described in Table S4. HCC4084 (blue arrow), was from tumor tissue specimen contains no tumor cells (Supplementary Table S1). SA3 (duplicated microarrays) and SA4 (green arrows) were tumor and non-malignant tissues from the same individual cultured under CRC conditions. SA6 and SA7 (orange arrows) were tumor and non-malignant tissues from the same individual cultured under CRC conditions.

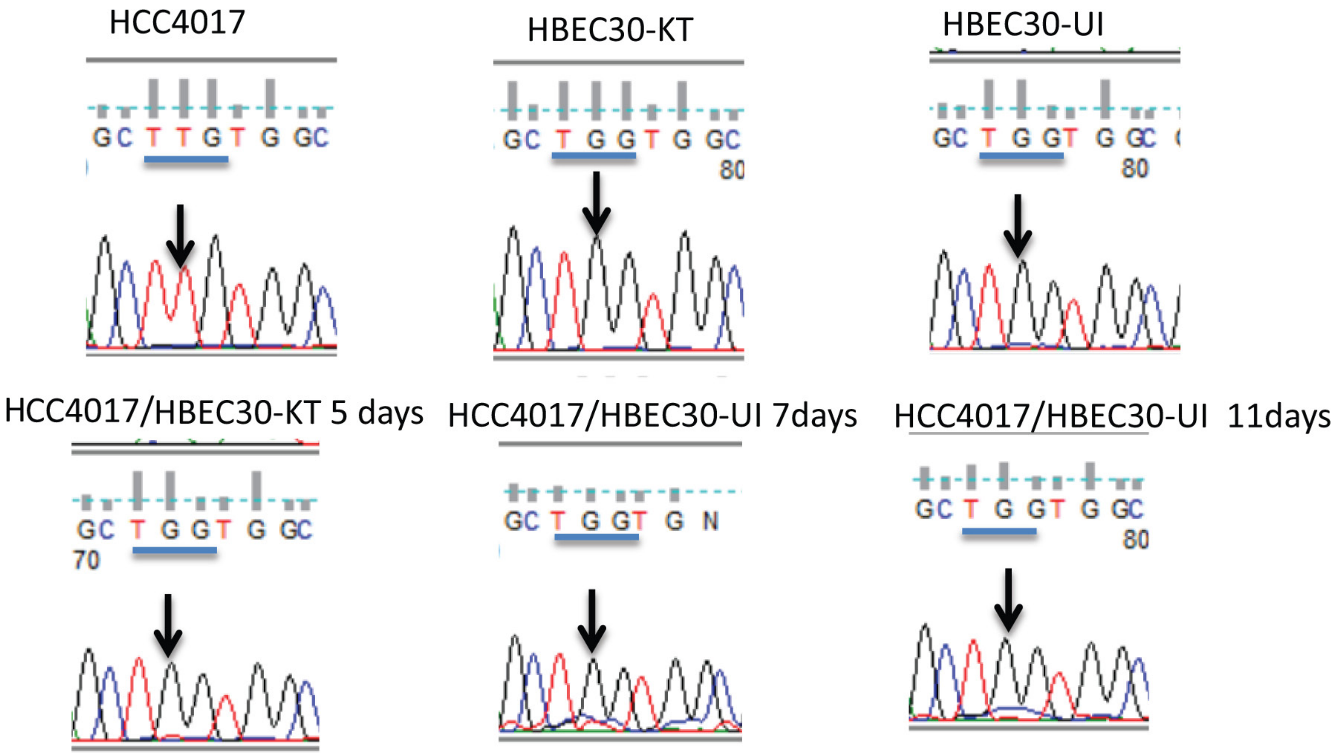


**Supplementary Figure S6: Sequencing of KRAS mutation in co-culture of HCC4017/HBEC30-KT and HCC4017/ HBEC30-UI.** HCC4017, HBEC30-KT and HBEC30-UI cells cultured alone (top panel), HCC4017/HBEC30-KT co-cultured for 5 days (left in lower panel), or HCC4017/HBEC30-UI co-cultured for seven or eleven days (middle and right in lower panel) were shown. HCC4017 carries KRAS mutation at codon 12 (TTG) with the allele frequency of 100% (top panel at left) while HBEC30-KT and HBEC30-UI carry wild type KRAS (TGG, top panel at middle and right). Arrows indicate the nucleotides sequence of KRAS where mutation occurs in HCC4017. Blue bars indicate the sequence of codon 12.

**Supplementary Table S1: Detailed patient information.** See Supplementary_Table_S1

**Supplementary Table S2: Information for non-malignant tissues used in CRC culture**

| Name | Age | Gender | Tissue type | Paired with | Source |
|---|---|---|---|---|---|
| HBEC67 | 65 | F | Bronchus | No | UTSW |
| HBEC78 | 54 | M | Bronchus | No | UTSW |
| HSAEC130 | 54 | M | Bronchus | No | UTSW |
| HBEC80 | 66 | M | Bronchus | No | UTSW |
| HSAEC132 | 66 | M | Peripheral lung | No | UTSW |
| HBEC81 | 59 | M | Bronchus | No | UTSW |
| HSAEC133 | 59 | M | Peripheral lung | No | UTSW |
| HSAEC137 | 61 | F | Peripheral lung | No | UTSW |
| HBEC4105 | 63 | F | Bronchus | No | UTSW |
| HBEC4106 | 63 | M | Bronchus | HSAEC4106 | UTSW |
| HSAEC4106 | 63 | M | Peripheral lung | HBEC4106 | UTSW |
| HBEC4108 | 44 | F | Bronchus | HCC4108/HSAEC4108 | UTSW |
| HSAEC4108 | 44 | F | Peripheral lung | HCC4108/HBEC4108 | UTSW |
| HSAEC4111 | 79 | F | Peripheral lung | No | UTSW |
| HBEC4119 | 69 | F | Bronchus | HCC4119/HSAEC4119 | UTSW |
| HSAEC4119 | 69 | F | Peripheral lung | HCC4119/HBEC4119 | UTSW |
| HBEC4163 | 47 | F | Bronchus | HSAEC4163 | UTSW |
| HSAEC4163 | 47 | F | Peripheral lung | HBEC4163 | UTSW |
| HBEC4164 | 80 | F | Bronchus | HSAEC4164 | UTSW |
| HSAEC4164 | 80 | F | Peripheral lung | HBEC4164 | UTSW |
| SA4 | 78 | F | Peripheral lung | SA3 | Yale |
| SA7 | 60 | M | Peripheral lung | SA6 | Yale |

**Supplementery Table S3: List of genes that were tested for mutations commonly mutated in non-small cell lung cancer**

| 1 | 2 | 3 |
|---|---|---|
| ALK | ERBB2 | CDKN2A |
| ARID1A | HLA-A | KEAP1 |
| BRAF | MLL2 | PIK3CA |
| EGFR | NFE2L2 | RB1 |
| HRAS | NOTCH1 | TP53 |
| KRAS | PTEN | |
| MAP2K1 | | |
| MET | | |
| MGA | | |
| NF1 | | |
| NRAS | | |
| RBM10 | | |
| RET | | |
| RIT1 | | |
| ROS1 | | |
| SETD2 | | |
| SMARCA4 | | |
| STK11 | | |
| U2AF1 | | |

Column 1: genes commonly mutated in adenocarcinoma. Column 2: genes commonly mutated in squamous cell carcinoma. Column 3: genes commonly mutated in both adenocarcinoma and squamous cell carcinoma.

**Supplementary Table S4: RNAs used in microarray studies.** See Supplementary_Table_S4

**Supplementary Table S5: Part A: Fifty most up regulated genes comparing CRC cultured tumor specimens with lung tumor cell lines and the comparison between non-malignant lung epithelial cells cultured under CRC or non-CRC condition with lung tumor cell lines**

| Symbol | CRC tumor vs Lung tumor cell lines (log2) | *T*-test *P* value | CRC normal vs Lung tumor cell lines (log2) | *T*-test *P* value | HREC-UI vs Lung tumor cell lines (log2) | *T*-test *P* value |
|---|---|---|---|---|---|---|
| KRT6A | 9.51 | 6.6E-78 | 9.76 | 3.8E-76 | 9.61 | 2.7E-75 |
| SCGB1A1 | 9.00 | 8.6E-28 | 2.94 | 5.7E-04 | 0.72 | 1.3E-04 |
| KRT5 | 8.66 | 2.9E-81 | 9.50 | 1.5E-85 | 9.33 | 1.2E-103 |
| LGALS7B | 8.32 | 2.3E-29 | 9.23 | 3.5E-26 | 8.16 | 1.6E-70 |
| KRT17 | 8.01 | 4.0E-57 | 8.40 | 7.1E-58 | 8.33 | 4.6E-57 |
| KRT13 | 8.00 | 3.9E-40 | 6.29 | 5.9E-10 | 2.75 | 6.8E-11 |
| LGALS7 | 7.79 | 4.2E-27 | 8.89 | 4.7E-24 | 7.85 | 4.5E-70 |
| SPRR1A | 7.49 | 1.9E-26 | 8.88 | 5.7E-23 | 6.25 | 1.1E-45 |
| SERPINB3 | 6.87 | 5.3E-26 | 6.25 | 2.1E-16 | 4.14 | 1.7E-35 |
| SERPINB13 | 6.82 | 1.7E-35 | 5.88 | 2.5E-19 | 4.34 | 9.2E-40 |
| COL17A1 | 6.70 | 1.8E-59 | 7.32 | 1.5E-51 | 7.02 | 1.6E-63 |
| KRT6B | 6.61 | 4.9E-22 | 8.64 | 2.7E-23 | 8.23 | 5.4E-100 |
| KRT6C | 6.57 | 1.3E-12 | 8.41 | 2.6E-11 | 7.97 | 3.2E-41 |
| CCND2 | 6.55 | 2.4E-42 | 6.51 | 2.2E-47 | 7.52 | 2.1E-60 |
| SERPINA3 | 6.54 | 1.3E-24 | 3.17 | 2.3E-07 | 5.01 | 6.9E-36 |
| KRT16 | 6.49 | 2.1E-43 | 7.93 | 1.2E-64 | 7.53 | 1.5E-95 |
| SPRR1B | 6.48 | 1.8E-24 | 6.97 | 1.4E-21 | 5.30 | 5.1E-45 |
| SERPINB2 | 6.44 | 1.8E-26 | 4.27 | 4.0E-12 | 5.19 | 4.4E-52 |
| HES2 | 6.33 | 4.8E-50 | 5.81 | 7.4E-40 | 6.58 | 2.5E-98 |
| DHRS9 | 6.29 | 1.7E-38 | 4.53 | 1.9E-17 | 2.84 | 1.4E-13 |
| S100A8 | 6.26 | 2.1E-22 | 7.42 | 1.2E-25 | 7.27 | 4.6E-60 |
| DSG3 | 6.26 | 1.0E-31 | 6.90 | 7.0E-31 | 6.07 | 1.6E-65 |
| KLK11 | 6.25 | 8.3E-32 | 6.78 | 1.7E-25 | 4.33 | 2.3E-37 |
| SPRR2D | 6.25 | 1.5E-20 | 6.45 | 1.2E-12 | 5.15 | 7.7E-37 |
| CLCA2 | 6.15 | 1.6E-36 | 7.21 | 1.9E-35 | 6.79 | 1.8E-103 |
| KRT14 | 6.09 | 6.2E-18 | 8.04 | 4.7E-16 | 7.81 | 1.6E-61 |
| S100A9 | 6.02 | 1.5E-32 | 5.92 | 1.4E-28 | 5.63 | 2.8E-31 |
| TP63 | 6.00 | 1.5E-55 | 6.73 | 8.5E-53 | 6.42 | 1.3E-58 |
| S100A2 | 5.99 | 1.2E-64 | 5.83 | 3.6E-50 | 5.83 | 2.1E-86 |
| CLCA4 | 5.96 | 1.2E-18 | 4.76 | 3.1E-11 | 2.11 | 3.1E-16 |
| ANXA8L2 | 5.93 | 3.8E-43 | 5.46 | 3.7E-26 | 5.71 | 1.6E-42 |
| SCGB3A1 | 5.91 | 3.4E-14 | 3.61 | 1.1E-04 | 1.38 | 3.2E-08 |
| SPRR3 | 5.89 | 3.1E-15 | 7.73 | 8.5E-16 | 3.47 | 2.1E-16 |
| GJB2 | 5.79 | 6.4E-35 | 6.60 | 8.7E-37 | 6.23 | 1.1E-65 |
| KRT4 | 5.78 | 1.1E-19 | 3.80 | 2.1E-06 | 0.07 | 8.1E-01 |
| FGFBP1 | 5.77 | 3.4E-36 | 5.74 | 5.8E-36 | 4.61 | 4.9E-33 |
| UGT1A1 | 5.66 | 2.1E-35 | 4.98 | 7.5E-21 | 4.34 | 4.2E-34 |
| PKP1 | 5.59 | 1.3E-28 | 6.95 | 8.4E-31 | 6.15 | 9.0E-98 |
| ANXA8L1 | 5.58 | 6.3E-41 | 4.67 | 3.2E-19 | 5.13 | 1.3E-44 |
| SERPINB5 | 5.55 | 8.2E-47 | 5.81 | 5.0E-48 | 5.77 | 6.6E-49 |
| CXCL1 | 5.54 | 1.0E-37 | 3.20 | 9.1E-12 | 3.73 | 1.1E-33 |
| ADH7 | 5.51 | 1.5E-18 | 5.72 | 6.8E-12 | 2.00 | 8.4E-14 |
| IL1RN | 5.50 | 8.0E-42 | 5.28 | 5.0E-23 | 5.08 | 1.7E-72 |
| SOX15 | 5.48 | 2.6E-53 | 6.12 | 4.4E-48 | 5.82 | 2.8E-59 |
| CCL20 | 5.32 | 1.9E-20 | 3.33 | 2.8E-12 | 4.99 | 3.6E-47 |
| DSC3 | 5.27 | 1.3E-58 | 5.90 | 6.3E-40 | 5.32 | 4.8E-80 |
| TGM1 | 5.26 | 2.3E-23 | 4.93 | 5.4E-16 | 3.70 | 1.0E-30 |
| AQP3 | 5.20 | 2.3E-21 | 4.13 | 1.5E-12 | 1.49 | 5.2E-14 |
| F3 | 5.19 | 1.7E-40 | 4.23 | 5.1E-22 | 5.21 | 9.9E-55 |
| PDPN | 5.15 | 2.0E-26 | 5.28 | 1.6E-29 | 6.42 | 5.2E-77 |

**Supplementary Table S5: Part B: Fifty most down regulated genes comparing CRC cultured tumor specimens with lung tumor cell lines and the comparison between non-malignant lung epithelial cells cultured under CRC or non-CRC condition with lung tumor cell lines**

| Symbol | CRC tumor vs Lung tumor cell lines (log2) | *T*-test *P* value | CRC normal vs Lung tumor cell lines (log2) | *T*-test *P* value | HREC-UI vs Lung tumor cell lines (log2) | *T*-test *P* value |
|---|---|---|---|---|---|---|
| FLJ22184 | -3.07 | 1.3E-24 | -2.86 | 1.3E-16 | -2.88 | 6.6E-33 |
| PNMA2 | -3.08 | 1.2E-19 | -3.44 | 2.3E-31 | -3.03 | 4.2E-27 |
| RAB3IL1 | –3.10 | 1.4E-17 | –2.87 | 4.5E-18 | –1.60 | 3.3E-13 |
| EPGN | –3.13 | 7.8E-79 | –3.11 | 5.0E-77 | –2.39 | 1.0E-22 |
| HOXD13 | –3.17 | 6.0E-82 | –3.17 | 4.3E-72 | –2.39 | 2.3E-20 |
| PRAME | –3.18 | 1.5E-26 | –2.92 | 2.0E-22 | –2.92 | 4.0E-24 |
| CPVL | –3.21 | 3.3E-16 | –3.07 | 2.9E-14 | –2.77 | 3.9E-19 |
| FLJ39632 | –3.21 | 2.6E-30 | –3.47 | 4.8E-42 | –3.44 | 2.4E-48 |
| MAP1B | –3.26 | 3.7E-10 | –3.33 | 5.0E-13 | –0.66 | 1.6E-03 |
| RAB6A | –3.26 | 4.1E-60 | –2.95 | 3.7E-20 | –1.92 | 1.3E-14 |
| HIST1H2BJ | –3.26 | 5.6E-26 | –3.53 | 3.4E-15 | –2.07 | 3.7E-14 |
| NUP210 | –3.27 | 1.3E-28 | –3.56 | 4.8E-62 | –3.49 | 8.0E-68 |
| HOXC6 | –3.27 | 1.1E-18 | –3.30 | 1.6E-25 | –3.37 | 3.1E-30 |
| MAFA | –3.28 | 3.1E-59 | –3.29 | 6.9E-59 | –2.74 | 2.2E-34 |
| FZD2 | –3.33 | 4.8E-15 | –3.46 | 4.3E-20 | –1.99 | 3.0E-18 |
| C3orf17 | –3.33 | 7.8E-47 | –3.25 | 1.4E-22 | –2.29 | 4.0E-12 |
| ZIC2 | –3.35 | 1.1E-16 | –3.83 | 1.2E-37 | –3.68 | 2.8E-37 |
| GPATCH1 | –3.41 | 2.7E-77 | –3.14 | 4.7E-27 | –2.59 | 3.4E-34 |
| PDLIM7 | –3.42 | 1.3E-28 | –3.64 | 2.9E-30 | –2.38 | 3.8E-17 |
| ARHGAP11A | –3.44 | 3.6E-101 | –3.39 | 3.4E-67 | –2.49 | 5.2E-18 |
| CARD9 | –3.45 | 3.8E-27 | –3.98 | 1.1E-51 | –3.00 | 6.3E-28 |
| FAM196A | –3.48 | 1.7E-73 | –3.61 | 4.4E–46 | -2.38 | 3.6E–12 |
| QPRT | –3.50 | 1.3E-11 | –4.85 | 2.3E-33 | –3.75 | 1.3E-22 |
| C13orf27 | –3.56 | 2.1E-23 | –3.65 | 6.6E-14 | –2.47 | 6.9E-17 |
| POFUT2 | –3.57 | 3.3E-66 | –3.44 | 7.2E-41 | –2.43 | 1.1E-12 |
| ACSL4 | –3.60 | 9.1E-93 | –3.51 | 1.8E-30 | –2.72 | 7.9E-21 |
| PTTG2 | –3.61 | 4.6E-49 | –3.62 | 5.0E-34 | –2.68 | 1.5E-15 |
| SCN3A | –3.65 | 8.5E-24 | –3.98 | 6.1E-17 | –3.22 | 3.1E-21 |
| PCBP4 | –3.68 | 6.9E-57 | –3.53 | 3.4E-28 | –2.55 | 1.9E-17 |
| PAX7 | –3.70 | 9.2E-78 | –3.20 | 3.4E-17 | –2.21 | 7.9E-16 |
| VGF | –3.74 | 1.3E-24 | –3.44 | 4.5E-21 | –3.16 | 1.9E-24 |
| DCDC5 | –3.77 | 4.8E-95 | –3.82 | 1.5E-87 | –2.86 | 4.8E-19 |
| GK2 | –3.78 | 3.7E-91 | –3.82 | 2.1E-57 | –2.87 | 7.8E-20 |
| FLJ41423 | –3.78 | 8.4E-73 | –3.84 | 4.0E-52 | –3.00 | 1.8E-23 |
| MUSK | –3.88 | 2.7E-82 | –3.88 | 9.5E-63 | –2.71 | 1.7E-13 |
| IGF2BP3 | –3.89 | 5.0E-16 | –4.83 | 4.2E-18 | –2.52 | 5.9E-24 |
| RNFT2 | –3.92 | 2.4E-35 | –4.57 | 2.3E-22 | –4.30 | 3.6E-75 |
| FAM69B | –3.93 | 3.5E-31 | –3.69 | 2.2E-18 | –3.51 | 5.0E-41 |
| RBPMS2 | –3.96 | 2.9E-24 | –4.71 | 1.1E-54 | –4.21 | 1.9E-50 |
| HOXB7 | –4.04 | 2.6E-18 | –4.74 | 3.4E-38 | –4.42 | 7.5E-36 |
| PRKCQ | –4.05 | 1.6E-38 | –3.77 | 4.0E-18 | –2.05 | 1.0E-12 |
| RTN2 | –4.20 | 2.1E-107 | –4.14 | 2.8E-59 | –3.14 | 1.0E-19 |
| GSTCD | –4.22 | 8.3E-91 | –4.18 | 1.9E-53 | –2.89 | 3.2E-14 |
| EXOG | –4.23 | 6.6E-47 | –4.07 | 2.0E-43 | –2.32 | 1.5E-06 |
| PTN | –4.24 | 1.4E-43 | –4.11 | 4.1E-37 | –3.18 | 7.3E-24 |
| MRPL55 | –4.32 | 8.3E-55 | –4.32 | 6.0E-29 | –3.11 | 1.7E-18 |
| SIRPG | –4.42 | 2.5E-93 | –4.45 | 1.0E-93 | –3.22 | 7.7E-17 |
| XAGE1A | –4.76 | 9.6E-17 | –5.15 | 2.2E-23 | –5.22 | 7.5E-24 |
| HTR2A | –5.50 | 8.4E-46 | –5.62 | 2.8E-92 | –4.41 | 7.2E-26 |
| EEF1A2 | –5.90 | 6.6E-24 | –5.95 | 1.3E-25 | –6.80 | 1.8E-66 |

## Supplementary Table S6: Summary of the CRC culture methods and reagents used at the four institutes

| Institution | UTSW | UTSW[a] (Tumor/normal co-culture) | Yale[a] | Moffitt | UC[a] |
|---|---|---|---|---|---|
| 3T3 strain used | NIH/3T3 | 3T3/J2 | 3T3/J2 | 3T3-Swiss albino | 3T3/J2 |
| Source of 3T3 | ATCC | Georgetown University | Georgetown University | ATCC | Kerafast Biotechnology |
| Medium used | ACL4 with 5% FBS | F medium with 5% FBS | F medium with 5% FBS | F medium with 5% FBS | F medium with 5% FBS |
| Enzyme digestion of the tumor tissue | No | N/A | Yes | Yes | Yes |
| Days of cells in CRC culture (range) | 25 (12–38) | 8 (5–11) | 19 (16–22) | 24 (15–34) | 25 (18,31) |

[a]Method used is the same as in the original CRC paper by Liu et al. N/A: not available.

## Supplementary Table S7: Summary of experiments and results from four centers

| Institution | UTSW | Yale | Moffitt | UC | Total |
|---|---|---|---|---|---|
| Number of lung tumor specimens | 20 | 17 | 9 | 2 | 48 |
| Number of mutations in tumor CRC culture/number of mutations in original tumor tissue | 1[a]/10 | 0/11 | 0/11 | 1[a]/2 | 2[a]/34 |
| CRC cultured tumor cells with aneuploid genome (CNV) | N/A | 0/9 | N/A | N/A | 0/9 |
| mRNA expression in CRC cultured tumors not clustered with non-malignant CRC cultured cells | 0/15 | 0/17 | N/A | N/A | 0/32 |
| Result of tumor/normal co-culture | Only normal cells present after 11 days | N/A | N/A | N/A | |

[a]Minor mutations detected in the samples. N/A: not available.
