# Supplementary material for: Non-malignant respiratory epithelial cells preferentially proliferate from resected non-small cell lung cancer specimens cultured under conditionally reprogrammed conditions
Source: Oncotarget. 2016 Dec 29;8(7):11114–26. doi: 10.18632/oncotarget.14366 (PMC5355251; doi:10.18632/oncotarget.14366)
Supplement: Supplementary file 3 [file oncotarget-08-11114-s003.docx]

**Table S4: RNAs used in microarray studies.**

| **Sort** | **Name** | **Type** | **Arrays** | | | | | | | |
| --- | --- | --- | --- | --- | --- | --- | --- | --- | --- | --- |
| 1 | A549 | Tumor Line | Illumina WG6-V3 | | | | | | | |
| 2 | Calu-1 | Tumor Line | Illumina WG6-V3 | | | | | | | |
| 3 | Calu-3 | Tumor Line | Illumina WG6-V3 | | | | | | | |
| 4 | Calu-6 | Tumor Line | Illumina WG6-V3 | | | | | | | |
| 5 | DFCI024 | Tumor Line | Illumina WG6-V3 | | | | | | | |
| 6 | DFCI032 | Tumor Line | Illumina WG6-V3 | | | | | | | |
| 7 | EKVX | Tumor Line | Illumina WG6-V3 | | | | | | | |
| 8 | NCI-H23 | Tumor Line | Illumina WG6-V3 | | | | | | | |
| 9 | NCI-H28 | Tumor Line | Illumina WG6-V3 | | | | | | | |
| 10 | NCI-H125 | Tumor Line | Illumina WG6-V3 | | | | | | | |
| 11 | H157 | Tumor Line | Illumina WG6-V3 | | | | | | | |
| 12 | NCI-H226 | Tumor Line | Illumina WG6-V3 | | | | | | | |
| 13 | NCI-H290 | Tumor Line | Illumina WG6-V3 | | | | | | | |
| 14 | NCI-H292 | Tumor Line | Illumina WG6-V3 | | | | | | | |
| 15 | NCI-H322 | Tumor Line | Illumina WG6-V3 | | | | | | | |
| 16 | NCI-H324 | Tumor Line | Illumina WG6-V3 | | | | | | | |
| 17 | NCI-H358 | Tumor Line | Illumina WG6-V3 | | | | | | | |
| 18 | NCI-H441 | Tumor Line | Illumina WG6-V3 | | | | | | | |
| 19 | NCI-H460 | Tumor Line | Illumina WG6-V3 | | | | | | | |
| 20 | NCI-H520 | Tumor Line | Illumina WG6-V3 | | | | | | | |
| 21 | NCI-H522 | Tumor Line | Illumina WG6-V3 | | | | | | | |
| 22 | NCI-H596 | Tumor Line | Illumina WG6-V3 | | | | | | | |
| 23 | NCI-H647 | Tumor Line | Illumina WG6-V3 | | | | | | | |
| 24 | NCI-H650 | Tumor Line | Illumina WG6-V3 | | | | | | | |
| 25 | NCI-H661 | Tumor Line | Illumina WG6-V3 | | | | | | | |
| 26 | NCI-H720 | Tumor Line | Illumina WG6-V3 | | | | | | | |
| 27 | NCI-H727 | Tumor Line | Illumina WG6-V3 | | | | | | | |
| 28 | NCI-H820 | Tumor Line | Illumina WG6-V3 | | | | | | | |
| 29 | NCI-H838 | Tumor Line | Illumina WG6-V3 | | | | | | | |
| 30 | NCI-H920 | Tumor Line | Illumina WG6-V3 | | | | | | | |
| 31 | NCI-H969 | Tumor Line | Illumina WG6-V3 | | | | | | | |
| 32 | NCI-H1155 | Tumor Line | Illumina WG6-V3 | | | | | | | |
| 33 | NCI-H1299 | Tumor Line | Illumina WG6-V3 | | | | | | | |
| 34 | NCI-H1355 | Tumor Line | Illumina WG6-V3 | | | | | | | |
| 35 | NCI-H1373 | Tumor Line | Illumina WG6-V3 | | | | | | | |
| 36 | NCI-H1385 | Tumor Line | Illumina WG6-V3 | | | | | | | |
| 37 | NCI-H1395 | Tumor Line | Illumina WG6-V3 | | | | | | | |
| 38 | NCI-H1437 | Tumor Line | Illumina WG6-V3 | | | | | | | |
| 39 | NCI-H1563 | Tumor Line | Illumina WG6-V3 | | | | | | | |
| 40 | NCI-H1568 | Tumor Line | Illumina WG6-V3 | | | | | | | |
| 41 | NCI-H1570 | Tumor Line | Illumina WG6-V3 | | | | | | | |
| 42 | NCI-H1573 | Tumor Line | Illumina WG6-V3 | | | | | | | |
| 43 | NCI-H1581 | Tumor Line | Illumina WG6-V3 | | | | | | | |
| 44 | NCI-H1648 | Tumor Line | Illumina WG6-V3 | | | | | | | |
| 45 | NCI-H1650 | Tumor Line | Illumina WG6-V3 | | | | | | | |
| 46 | NCI-H1651 | Tumor Line | Illumina WG6-V3 | | | | | | | |
| 47 | NCI-H1666 | Tumor Line | Illumina WG6-V3 | | | | | | | |
| 48 | NCI-H1693 | Tumor Line | Illumina WG6-V3 | | | | | | | |
| 49 | NCI-H1703 | Tumor Line | Illumina WG6-V3 | | | | | | | |
| 50 | NCI-H1734 | Tumor Line | Illumina WG6-V3 | | | | | | | |
| 51 | NCI-H1755 | Tumor Line | Illumina WG6-V3 | | | | | | | |
| 52 | NCI-H1781 | Tumor Line | Illumina WG6-V3 | | | | | | | |
| 53 | NCI-H1792 | Tumor Line | Illumina WG6-V3 | | | | | | | |
| 54 | NCI-H1793 | Tumor Line | Illumina WG6-V3 | | | | | | | |
| 55 | NCI-H1819 | Tumor Line | Illumina WG6-V3 | | | | | | | |
| 56 | NCI-H1838 | Tumor Line | Illumina WG6-V3 | | | | | | | |
| 57 | NCI-H1869 | Tumor Line | Illumina WG6-V3 | | | | | | | |
| 58 | NCI-H1944 | Tumor Line | Illumina WG6-V3 | | | | | | | |
| 59 | NCI-H1975 | Tumor Line | Illumina WG6-V3 | | | | | | | |
| 60 | NCI-H1993 | Tumor Line | Illumina WG6-V3 | | | | | | | |
| 61 | NCI-H2009 | Tumor Line | Illumina WG6-V3 | | | | | | | |
| 62 | NCI-H2023 | Tumor Line | Illumina WG6-V3 | | | | | | | |
| 63 | NCI-H2030 | Tumor Line | Illumina WG6-V3 | | | | | | | |
| 64 | NCI-H2052 | Tumor Line | Illumina WG6-V3 | | | | | | | |
| 65 | NCI-H2073 | Tumor Line | Illumina WG6-V3 | | | | | | | |
| 66 | NCI-H2085 | Tumor Line | Illumina WG6-V3 | | | | | | | |
| 67 | NCI-H2086 | Tumor Line | Illumina WG6-V3 | | | | | | | |
| 68 | NCI-H2087 | Tumor Line | Illumina WG6-V3 | | | | | | | |
| 69 | NCI-H2106 | Tumor Line | Illumina WG6-V3 | | | | | | | |
| 70 | NCI-H2122 | Tumor Line | Illumina WG6-V3 | | | | | | | |
| 71 | NCI-H2126 | Tumor Line | Illumina WG6-V3 | | | | | | | |
| 72 | NCI-H2170 | Tumor Line | Illumina WG6-V3 | | | | | | | |
| 73 | NCI-H2172 | Tumor Line | Illumina WG6-V3 | | | | | | | |
| 74 | NCI-H2228 | Tumor Line | Illumina WG6-V3 | | | | | | | |
| 75 | NCI-H2250 | Tumor Line | Illumina WG6-V3 | | | | | | | |
| 76 | NCI-H2258 | Tumor Line | Illumina WG6-V3 | | | | | | | |
| 77 | NCI-H2291 | Tumor Line | Illumina WG6-V3 | | | | | | | |
| 78 | NCI-H2342 | Tumor Line | Illumina WG6-V3 | | | | | | | |
| 79 | NCI-H2347 | Tumor Line | Illumina WG6-V3 | | | | | | | |
| 80 | NCI-H2405 | Tumor Line | Illumina WG6-V3 | | | | | | | |
| 81 | NCI-H2882 | Tumor Line | Illumina WG6-V3 | | | | | | | |
| 82 | NCI-H2887 | Tumor Line | Illumina WG6-V3 | | | | | | | |
| 83 | NCI-H3122 | Tumor Line | Illumina WG6-V3 | | | | | | | |
| 84 | NCI-H3255 | Tumor Line | Illumina WG6-V3 | | | | | | | |
| 85 | HCC15 | Tumor Line | Illumina WG6-V3 | | | | | | | |
| 86 | HCC44 | Tumor Line | Illumina WG6-V3 | | | | | | | |
| 87 | HCC78 | Tumor Line | Illumina WG6-V3 | | | | | | | |
| 88 | HCC95 | Tumor Line | Illumina WG6-V3 | | | | | | | |
| 89 | HCC193 | Tumor Line | Illumina WG6-V3 | | | | | | | |
| 90 | HCC364 | Tumor Line | Illumina WG6-V3 | | | | | | | |
| 91 | HCC366 | Tumor Line | Illumina WG6-V3 | | | | | | | |
| 92 | HCC461 | Tumor Line | Illumina WG6-V3 | | | | | | | |
| 93 | HCC515 | Tumor Line | Illumina WG6-V3 | | | | | | | |
| 94 | HCC827 | Tumor Line | Illumina WG6-V3 | | | | | | | |
| 95 | HCC1171 | Tumor Line | Illumina WG6-V3 | | | | | | | |
| 96 | HCC1195 | Tumor Line | Illumina WG6-V3 | | | | | | | |
| 97 | HCC1313 | Tumor Line | Illumina WG6-V3 | | | | | | | |
| 98 | HCC1359 | Tumor Line | Illumina WG6-V3 | | | | | | | |
| 99 | HCC1438 | Tumor Line | Illumina WG6-V3 | | | | | | | |
| 100 | HCC1833 | Tumor Line | Illumina WG6-V3 | | | | | | | |
| 101 | HCC1897 | Tumor Line | Illumina WG6-V3 | | | | | | | |
| 102 | HCC2108 | Tumor Line | Illumina WG6-V3 | | | | | | | |
| 103 | HCC2279 | Tumor Line | Illumina WG6-V3 | | | | | | | |
| 104 | HCC2374 | Tumor Line | Illumina WG6-V3 | | | | | | | |
| 105 | HCC2429 | Tumor Line | Illumina WG6-V3 | | | | | | | |
| 106 | HCC2450 | Tumor Line | Illumina WG6-V3 | | | | | | | |
| 107 | HCC2814 | Tumor Line | Illumina WG6-V3 | | | | | | | |
| 108 | HCC2935 | Tumor Line | Illumina WG6-V3 | | | | | | | |
| 109 | HCC3051 | Tumor Line | Illumina WG6-V3 | | | | | | | |
| 110 | HCC4006 | Tumor Line | Illumina WG6-V3 | | | | | | | |
| 111 | HCC4011 | Tumor Line | Illumina WG6-V3 | | | | | | | |
| 112 | HCC4017 | Tumor Line | Illumina WG6-V3 | | | | | | | |
| 113 | HCC4018 | Tumor Line | Illumina WG6-V3 | | | | | | | |
| 114 | HCC4019 | Tumor Line | Illumina WG6-V3 | | | | | | | |
| 115 | HCC4153 | Tumor Line | Illumina HT12-V4 | | | | | | | |
| 116 | HOP-62 | Tumor Line | Illumina WG6-V3 | | | | | | | |
| 117 | HOP-92 | Tumor Line | Illumina WG6-V3 | | | | | | | |
| 118 | PC-9 | Tumor Line | Illumina WG6-V3 | | | | | | | |
| 119 | SK-LU-1 | Tumor Line | Illumina WG6-V3 | | | | | | | |
| 120 | HBEC3-UI | HREC | Illumina WG6-V3 | | | | | | | |
| 121 | HBEC24-UI | HREC | Illumina HT12-V4 | | | | | | | |
| 122 | HBEC29-UI | HREC | Illumina HT12-V4 | | | | | | | |
| 123 | HBEC35-UI | HREC | Illumina HT12-V4 | | | | | | | |
| 124 | HBEC38-UI | HREC | Illumina HT12-V4 | | | | | | | |
| 125 | HBEC39-UI | HREC | Illumina HT12-V4 | | | | | | | |
| 126 | HBEC51-UI | HREC | Illumina HT12-V4 | | | | | | | |
| 127 | HBEC52-UI | HREC | Illumina HT12-V4 | | | | | | | |
| 128 | HBEC55-UI | HREC | Illumina HT12-V4 | | | | | | | |
| 129 | HBEC66-UI | HREC | Illumina HT12-V4 | | | | | | | |
| 130 | HBEC67-UI | HREC | Illumina HT12-V4 | | | | | | | |
| 131 | HBEC70-UI | HREC | Illumina HT12-V4 | | | | | | | |
| 132 | HBEC75-UI | HREC | Illumina HT12-V4 | | | | | | | |
| 133 | HBEC76-UI | HREC | Illumina HT12-V4 | | | | | | | |
| 134 | HBEC77-UI | HREC | Illumina HT12-V4 | | | | | | | |
| 135 | HBEC78-UI | HREC | Illumina HT12-V4 | | | | | | | |
| 136 | HBEC80-UI | HREC | Illumina HT12-V4 | | | | | | | |
| 137 | HBEC81-UI | HREC | Illumina HT12-V4 | | | | | | | |
| 138 | HBEC82-UI | HREC | Illumina HT12-V4 | | | | | | | |
| 139 | HBEC4105-UI | HREC | Illumina HT12-V4 | | | | | | | |
| 140 | HBEC4106-UI | HREC | Illumina HT12-V4 | | | | | | | |
| 141 | HBEC4144-UI | HREC | Illumina HT12-V4 | | | | | | | |
| 142 | HBEC4152-UI | HREC | Illumina HT12-V4 | | | | | | | |
| 143 | HBEC4154-UI | HREC | Illumina HT12-V4 | | | | | | | |
| 144 | HBEC4157-UI | HREC | Illumina HT12-V4 | | | | | | | |
| 145 | HSAEC1-UI | HREC | Illumina WG6-V3 | | | | | | | |
| 146 | HSAEC2-UI | HREC | Illumina WG6-V3 | | | | | | | |
| 147 | HSAEC13-UI | HREC | Illumina WG6-V3 | | | | | | | |
| 148 | HSAEC15-UI | HREC | Illumina WG6-V3 | | | | | | | |
| 149 | HSAEC22-UI | HREC | Illumina WG6-V3 | | | | | | | |
| 150 | HSAEC24-UI | HREC | Illumina WG6-V3 | | | | | | | |
| 151 | HSAEC30-UI | HREC | Illumina WG6-V3 | | | | | | | |
| 152 | HSAEC31-UI | HREC | Illumina WG6-V3 | | | | | | | |
| 153 | HSAEC35-UI | HREC | Illumina WG6-V3 | | | | | | | |
| 154 | HSAEC36-UI | HREC | Illumina WG6-V3 | | | | | | | |
| 155 | HSAEC37-UI | HREC | Illumina WG6-V3 | | | | | | | |
| 156 | HSAEC38-UI | HREC | Illumina WG6-V3 | | | | | | | |
| 157 | HSAEC45-UI | HREC | Illumina WG6-V3 | | | | | | | |
| 158 | HSAEC49-UI | HREC | Illumina WG6-V3 | | | | | | | |
| 159 | HSAEC75-UI | HREC | Illumina HT12-V4 | | | | | | | |
| 160 | HSAEC78-UI | HREC | Illumina HT12-V4 | | | | | | | |
| 161 | HSAEC81-UI | HREC | Illumina HT12-V4 | | | | | | | |
| 162 | HSAEC82-UI | HREC | Illumina HT12-V4 | | | | | | | |
| 163 | HSAEC86-UI | HREC | Illumina HT12-V4 | | | | | | | |
| 164 | HSAEC88-UI | HREC | Illumina HT12-V4 | | | | | | | |
| 165 | HSAEC97-UI | HREC | Illumina HT12-V4 | | | | | | | |
| 166 | HSAEC101-UI | HREC | Illumina HT12-V4 | | | | | | | |
| 167 | HSAEC106-UI | HREC | Illumina HT12-V4 | | | | | | | |
| 168 | HSAEC114-UI | HREC | Illumina HT12-V4 | | | | | | | |
| 169 | HSAEC121-UI | HREC | Illumina HT12-V4 | | | | | | | |
| 170 | HSAEC127-UI | HREC | Illumina HT12-V4 | | | | | | | |
| 171 | HSAEC128-UI | HREC | Illumina HT12-V4 | | | | | | | |
| 172 | HSAEC123-UI | HREC | Illumina HT12-V4 | | | | | | | |
| 173 | HSAEC125-UI | HREC | Illumina HT12-V4 | | | | | | | |
| 174 | HSAEC130-UI | HREC | Illumina HT12-V4 | | | | | | | |
| 175 | HSAEC132-UI | HREC | Illumina HT12-V4 | | | | | | | |
| 176 | HSAEC136-UI | HREC | Illumina HT12-V4 | | | | | | | |
| 177 | HSAEC137-UI | HREC | Illumina HT12-V4 | | | | | | | |
| 178 | HSAEC142-UI | HREC | Illumina HT12-V4 | | | | | | | |
| 179 | HSAEC4106-UI | HREC | Illumina HT12-V4 | | | | | | | |
| 180 | HSAEC4112-UI | HREC | Illumina HT12-V4 | | | | | | | |
| 181 | HSAEC4118-UI | HREC | Illumina HT12-V4 | | | | | | | |
| 182 | HSAEC4121-UI | HREC | Illumina HT12-V4 | | | | | | | |
| 183 | HSAEC4128-UI | HREC | Illumina HT12-V4 | | | | | | | |
| 184 | HSAEC4130-UI | HREC | Illumina HT12-V4 | | | | | | | |
| 185 | HSAEC4155-UI | HREC | Illumina HT12-V4 | | | | | | | |
| 186 | HBEC67-CRC | CRC-Normal | Illumina HT12-V4 | | | | | | | |
| 187 | HBEC78-CRC | CRC-Normal | Illumina HT12-V4 | | | | | | | |
| 188 | HBEC81-CRC | CRC-Normal | Illumina HT12-V4 | | | | | | | |
| 189 | HBEC80-CRC | CRC-Normal | Illumina HT12-V4 | | | | | | | |
| 190 | HBEC4105-CRC | CRC-Normal | Illumina HT12-V4 | | | | | | | |
| 191 | HBEC4106-CRC | CRC-Normal | Illumina HT12-V4 | | | | | | | |
| 192 | HBEC4108-CRC | CRC-Normal | Illumina HT12-V4 | | | | | | | |
| 193 | HBEC4119-CRC | CRC-Normal | Illumina HT12-V4 | | | | | | | |
| 194 | HSAEC130-CRC | CRC-Normal | Illumina HT12-V4 | | | | | | | |
| 195 | HSAEC132-CRC | CRC-Normal | Illumina HT12-V4 | | | | | | | |
| 196 | HSAEC133-CRC | CRC-Normal | Illumina HT12-V4 | | | | | | | |
| 197 | HSAEC137-CRC | CRC-Normal | Illumina HT12-V4 | | | | | | | |
| 198 | HSAEC4106-CRC | CRC-Normal | Illumina HT12-V4 | | | | | | | |
| 199 | HSAEC4108-CRC | CRC-Normal | Illumina HT12-V4 | | | | | | | |
| 200 | HSAEC4111-CRC | CRC-Normal | Illumina HT12-V4 | | | | | | | |
| 201 | HSAEC4119-CRC | CRC-Normal | Illumina HT12-V4 | | | | | | | |
| 202 | HCC4068-CRC | CRC-Tumor UTSW | Illumina HT12-V4 | | | | | | | |
| 203 | HCC4074-CRC | CRC-Tumor UTSW | Illumina HT12-V4 | | | | | | | |
| 204 | HCC4080-CRC | CRC-Tumor UTSW | Illumina HT12-V4 | | | | | | | |
| 205 | HCC4079-CRC | CRC-Tumor UTSW | Illumina HT12-V4 | | | | | | | |
| 206 | HCC4082-CRC | CRC-Tumor UTSW | Illumina HT12-V4 | | | | | | | |
| 207 | HCC4083-CRC | CRC-Tumor UTSW | Illumina HT12-V4 | | | | | | | |
| 208 | HCC4084-CRC* | CRC-Normal | Illumina HT12-V4 | | | | | | | |
| 209 | HCC4085-CRC | CRC-Tumor UTSW | Illumina HT12-V4 | | | | | | | |
| 210 | HCC4086-CRC | CRC-Tumor UTSW | Illumina HT12-V4 | | | | | | | |
| 211 | HCC4087-CRC | CRC-Tumor UTSW | Illumina HT12-V4 | | | | | | | |
| 212 | HCC4088-CRC | CRC-Tumor UTSW | Illumina HT12-V4 | | | | | | | |
| 213 | HCC4114-CRC | CRC-Tumor UTSW | Illumina HT12-V4 | | | | | | | |
| 214 | HCC4115-CRC | CRC-Tumor UTSW | Illumina HT12-V4 | | | | | | | |
| 215 | HCC4117-CRC | CRC-Tumor UTSW | Illumina HT12-V4 | | | | | | | |
| 216 | HCC4120-CRC | CRC-Tumor UTSW | Illumina HT12-V4 | | | | | | | |
| 217 | SA3_1 | CRC-Tumor Yale | Illumina HT12-V4 | | | | | | | |
| 218 | SA3_2 | CRC-Tumor Yale | Illumina HT12-V4 | | | | | | | |
| 219 | SA4 | CRC-Normal | Illumina HT12-V4 | | | | | | | |
| 220 | SA6 | CRC-Tumor Yale | Illumina HT12-V4 | | | | | | | |
| 221 | SA7 | CRC-Normal | Illumina HT12-V4 | | | | | | | |
| 222 | SA8 | CRC-Tumor Yale | Illumina HT12-V4 | | | | | | | |
| 223 | SA9 | CRC-Tumor Yale | Illumina HT12-V4 | | | | | | | |
| 224 | SA11 | CRC-Tumor Yale | Illumina HT12-V4 | | | | | | | |
| 225 | SA14 | CRC-Tumor Yale | Illumina HT12-V4 | | | | | | | |
| 226 | SA15 | CRC-Tumor Yale | Illumina HT12-V4 | | | | | | | |
| 227 | SA33 | CRC-Tumor Yale | Illumina HT12-V4 | | | | | | | |
| 228 | SA45 | CRC-Tumor Yale | Illumina HT12-V4 | | | | | | | |
| 229 | SA59 | CRC-Tumor Yale | Illumina HT12-V4 | | | | | | | |
| 230 | SA60 | CRC-Tumor Yale | Illumina HT12-V4 | | | | | | | |
| 231 | SA70 | CRC-Tumor Yale | Illumina HT12-V4 | | | | | | | |
| 232 | SA75 | CRC-Tumor Yale | Illumina HT12-V4 | | | | | | | |
| 233 | SA76 | CRC-Tumor Yale | Illumina HT12-V4 | | | | | | | |
| 234 | SA77 | CRC-Tumor Yale | Illumina HT12-V4 | | | | | | | |
| 235 | SA80 | CRC-Tumor Yale | Illumina HT12-V4 | | | | | | | |
| 236 | SA83 | CRC-Tumor Yale | Illumina HT12-V4 | | | | | | | |
|  | | | | | | | | |  |  |
|  | | |  |  |  |  |  |  |  |  |
|  | | |  |  |  |  |  |  |  |  |
|  | | |  |  |  |  |  |  |  |  |
|  | | |  |  |  |  |  |  |  |  |
|  | | |  |  |  |  |  |  |  |  |
|  | | |  |  |  |  |  |  |  |  |
|  | | |  |  |  |  |  |  |  |  |
|  | | |  |  |  |  |  |  |  |  |
